# Affinity and costimulatory architecture-associated toxicity in mesothelin-targeted TCR-mimetic CAR-T cells

**DOI:** 10.3389/fimmu.2026.1947351

**Published:** 2026-09-24

**Authors:** Wei Du, Siqi Li, Shaomei Gan, Chen Wang, Rui Lu, Wenbin Yu, Jie Lin, Xidai Long, Fang Tang, Enxiu Wang

**Affiliations:** 1 Clinical Pathological Diagnosis & Research Center, Youjiang Medical University for Nationalities, Baise, China; 2 Nanjing CART Medical Technology Co. Ltd., Nanjing, China; 3 School of Life Science and Technology, China Pharmaceutical University, Nanjing, China; 4 Department of Pathology, The Affiliated Hospital of Youjiang Medical University for Nationalities, Baise, China; 5 The Key Laboratory of Molecular Pathology (Hepatobiliary Diseases) of Guangxi, Baise, China; 6 School of Modern Pharmaceutical Industry, China Pharmaceutical University, Nanjing, China

**Keywords:** 4-1BB, antibody affinity, CAR-T toxicity, mesothelin, TCR-mimetic CAR-T

## Abstract

**Introduction:**

TCR-mimetic CAR-T combines antibody-based targeting with physiological TCR signaling, yet suboptimal co-stimulation and safety risks remain key barriers for solid tumor therapy. The prevailing assumption that stronger co-stimulation yields superior efficacy has not been systematically tested in TCR-mimetic CAR-T architectures.

**Methods:**

We developed a Mesothelin-targeted scFv-CD3ϵ TCR-mimetic CAR-T platform and systematically compared co-stimulatory modules—4-1BB, BRM-truncated 4-1BB^ΔBRM^, and the 80BB chimera—alongside CD3ζ overexpression. To define the requirements for toxicity, we generated a series of control constructs and compared SS1 versus MOR-based scFvs targeting the same antigen, which differ in their binding properties including affinity.

**Results:**

The optimized SS1-ϵζ-80BB TCR-mimetic CAR-T exhibited potent anti-tumor activity but unexpectedly induced dose-dependent lethal toxicity. Systematic controls identified the intact 80BB chimera as the construct associated with lethality. Deleting the 4-1BB intracellular domain, rerouting the 4-1BB intracellular domain to an antigen-dependent format, or replacing SS1 with MOR each abrogated toxicity while preserving anti-tumor efficacy. The SS1-ϵζ-80BB architecture was associated with excessive T-cell expansion and multi-organ infiltration while the MOR-ϵζ-80BB architecture was moderate.

**Discussion:**

These observations suggest that the mode of co-stimulatory configuration, rather than its mere presence, influences safety outcomes in TCR-mimetic CAR-T cells. Modifying either the affinity-related properties or the 80BB-based architecture preserved efficacy while reducing toxicity, providing potential design considerations for affinity selection and co-stimulator design in the mesothelin-targeted TCR-mimetic CAR-T platform.

## Introduction

Chimeric antigen receptor (CAR) T cells have achieved revolutionary success in hematologic malignancies, but their efficacy against solid tumors remains limited (1, 2). To address this challenge, various platforms that mimic the physiological T-cell receptor (TCR) complex have been developed. They are collectively termed TCR-mimetic CARs. Representative architectures include T-cell antigen couplers (TAC) (3), antibody-TCR (AbTCR) (4), TCR fusion constructs (TRuC) (5, 6), synthetic TCR and antigen receptor (STAR) (7), and HLA-independent TCR (HIT) (8).

Although these platforms support physiological TCR signaling, they lack dedicated co-stimulatory modules, leading to insufficient activation and rapid exhaustion (9, 10). In addition, limited expression of endogenous CD3 subunits, particularly CD3ζ, limits the surface expression of engineered receptors by restricting the availability of functional TCR complexes (11–13).

To improve co-stimulation, direct fusion of 4-1BB or CD28 intracellular domains to CD3 chains has been explored (14–17), along with other innovative approaches such as membrane-anchored IL-15 co-expression (18), autocrine IL-7/CCL21 co-expression (19), and PD-1-CD28 chimeric switch receptors (20). Lah et al. (21) demonstrated that, when 4-1BB is fused directly to CD3ζ, the basic residue-rich motif (BRM) in the ICD of full-length 4-1BB disrupts TCR complex assembly. In contrast, BRM-truncated 4-1BB^ΔBRM^ preserves co-stimulation without impairing TCR function. Dobrin et al. (8) developed 80BB, a chimeric co-stimulatory receptor consisting of the CD80 ectodomain fused to the 4-1BB endodomain, which delivers antigen-independent CD28 and 4-1BB signaling through cell-surface CD28/CTLA-4 engagement and enhances metabolic fitness, expansion, and anti-tumor efficacy in HIT and TCR-engineered T cells. While Dobrin et al. reported the efficacy and mechanism of 80BB in HIT and TCR-T platforms, its safety profile in scFv-CD3ϵ-based TCR-mimetic CAR-T architectures had not been systematically evaluated.

Beyond co-stimulation, proper TCR surface density depends on coordinated expression of CD3 subunits (22–24). TCR surface density directly influences functional avidity (25–27), and CD3ζ has been reported as rate-limiting—its down-regulation impairs T-cell function (26, 28), whereas forced expression enhances TCR surface levels and antigen-specific function (29). We therefore examined whether enhancing both TCR surface expression and co-stimulation could improve functional outcomes in the TCR-mimetic CAR-T platform, while recognizing that such enhanced signaling might carry unanticipated safety risks.

Building on these advances, we performed stepwise optimization in an scFv-CD3ϵ-based TCR-mimetic CAR targeting mesothelin (MSLN). We first compared antigen-dependent co-stimulatory modules (4-1BB vs 4-1BB^ΔBRM^), then directly compared 4-1BB^ΔBRM^ with the antigen-independent 80BB chimera, and finally evaluated the effect of CD3 subunit (γ, δ, ζ) on TCR-mimetic CAR surface expression.

Our results showed that the 80BB chimera provided superior functional activity compared with 4-1BB^ΔBRM^, and that CD3ζ overexpression further enhanced TCR-mimetic CAR surface expression and functional activity. Unexpectedly, the fully optimized SS1-ϵζ-80BB TCR-mimetic CAR induced dose-dependent lethal toxicity in mouse tumor models despite potent anti-tumor efficacy. We compared two scFvs, SS1 and MOR, which differ in their binding properties including affinity, to assess whether the observed 80BB phenotype was scFv-dependent. Deleting the 4-1BB intracellular domain, rerouting the 4-1BB intracellular domain to an antigen-dependent format, or replacing SS1 with MOR each abrogated toxicity while preserving anti-tumor efficacy. These findings suggest that balancing scFv properties and co-stimulatory configuration may be important for modulating the safety profile of mesothelin-targeted TCR-mimetic CAR-T cells, and provide potential design considerations for affinity selection and co-stimulation design in this platform.

## Results

### 4-1BB^ΔBRM^ enhances antigen-dependent TCR-mimetic CAR function *in vitro*

To evaluate the functional impact of the BRM motif of 4-1BB on TCR-mimetic CAR-T, we constructed MSLN-targeted TCR-mimetic CAR. We fused the SS1 scFv to the N-terminus of CD3ϵ. Then, we fused either the full-length intracellular domain of 4-1BB (ϵζ-BB) or the BRM-deleted intracellular domain of 4-1BB (ϵζ-BB^ΔBRM^) to the C-terminus of CD3ζ (**Figures 1A, B**). Surface expression of TCR-mimetic CAR-T was comparable among groups (**Supplementary Figures S1A–C**). MSLN expression on SKOV3 target cells was confirmed by flow cytometry (**Supplementary Figure S1D**). Short-term cytotoxicity was also similar across all groups (**Supplementary Figure S1E**). Compared with the ϵζ control, SS1-ϵζ-BB^ΔBRM^ induced higher IFN-γ and IL-2 secretion upon antigen stimulation. In contrast, SS1-ϵζ-BB reduced cytokine production (**Figures 1C, D**). These results align with observations by Lah et al. (21) in which the BRM motif of 4-1BB impairs TCR function. Co-stimulatory benefits were observed upon BRM removal.

**Figure 1:**
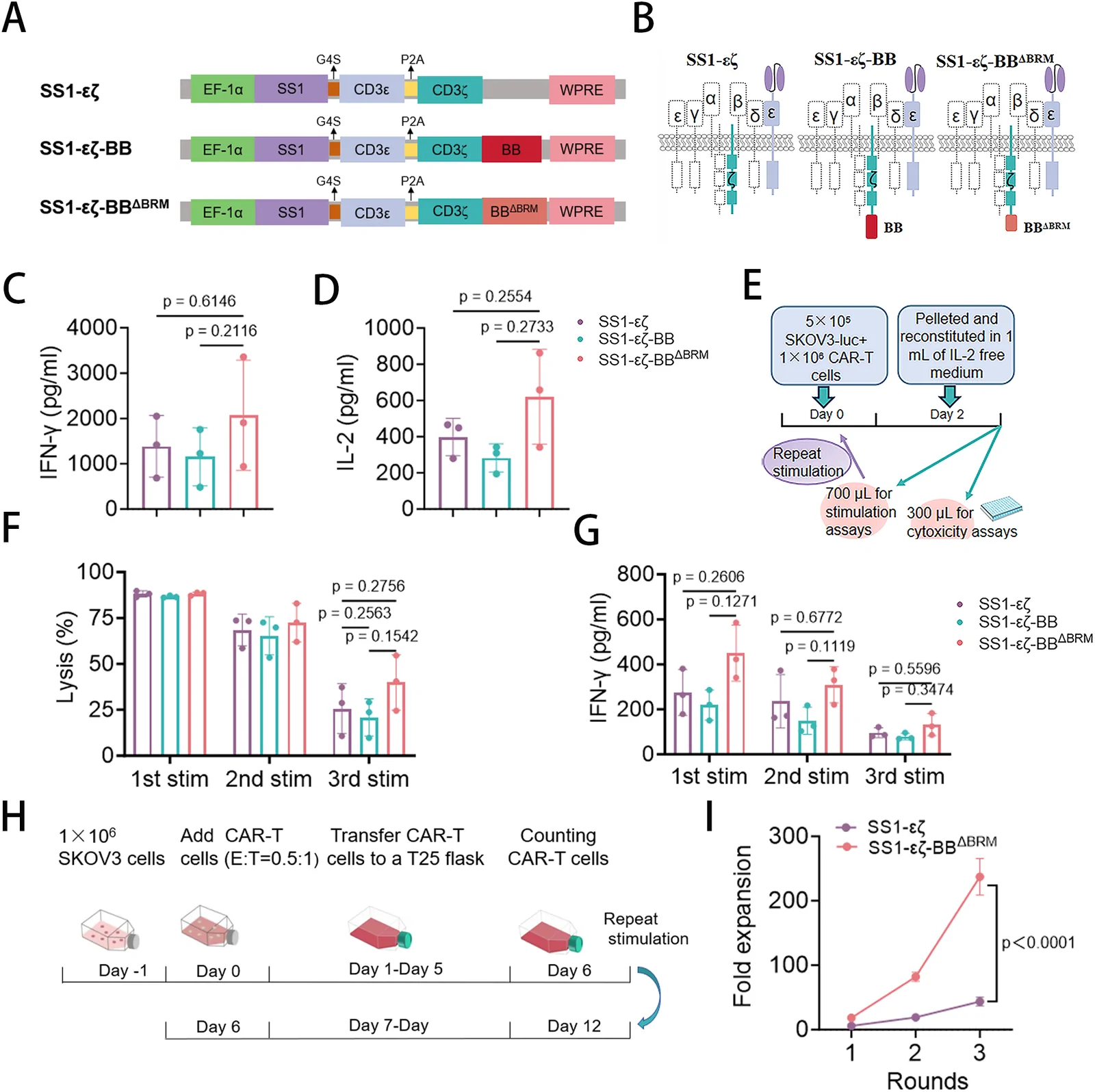
4-1BB^ΔBRM^ enhances TCR-mimetic CAR-T-T cell persistence and function *in vitro*. **(A)** Schematic diagram of lentiviral vectors: SS1-ϵζ (CD3ζ only), SS1-ϵζ-BB (CD3ζ + 4-1BB ICD fusion), and SS1-ϵζ-BB^ΔBRM^ (CD3ζ + BRM truncated 4-1BB ICD fusion). **(B)** Schematic representation of SS1-ϵζ, SS1-ϵζ-BB, and SS1-ϵζ-BB^ΔBRM^ constructs within the TCR complex. **(C, D)** IFN-γ **(C)** and IL-2 **(D)** concentrations in the supernatant 24 h after co-culture with MSLN^+^ SKOV3-luc target cells at an effector-to-target (E:T) ratio of 2:1. Data are from three independent donors, each shown as a single data point after averaging technical replicates (n = 3). *p* values were calculated by one-way ANOVA. **(E–G)** Schematic of the serial killing assay **(E)**, cytotoxic activity after each stimulation cycle **(F)**, and IFN-γ production in the supernatant **(G)**. Data are from three independent donors, each shown as a single data point after averaging technical replicates (n = 3). *p* values were calculated by two-way ANOVA. **(H, I)** Schematic of the antigen-stimulated expansion assay **(H)** and cumulative fold expansion of CAR-T cells over time **(I)**. Data are from three experimental replicates from one representative donor. Two-way repeated-measures ANOVA was performed for descriptive purposes; the data should be interpreted as exploratory. Data have been presented as mean ± SD.

In long term antigen exposure assays, SS1-ϵζ-BB^ΔBRM^ TCR-mimetic CAR-T cells showed more durable cytotoxicity (**Figures 1E, F**) and higher IFN-γ secretion (**Figure 1G**). It also exhibited a trend of greater proliferation under repeated antigen stimulation (**Figure 1H, I**). Thus, 4-1BB^ΔBRM^ enhances the functional persistence of TCR-mimetic CAR T cells. It was selected as the benchmark antigen-dependent co-stimulatory module.

### 80BB co-stimulation is superior to 4-1BB^ΔBRM^ in TCR-mimetic CAR-T

Although 4-1BB^ΔBRM^ improved function *in vitro*, its antigen dependence may limit long-term persistence. Dobrin et al. (8) reported that the antigen-independent chimera 80BB enhances TCR-T cell function. We therefore generated SS1-ϵζ-80BB TCR-mimetic CAR with matched CD3ζ overexpression relative to SS1-ϵζ-BB^ΔBRM^ (**Figures 2A, B**). SS1-ϵζ-80BB mediated higher cytotoxicity (**Figure 2C**) and elevated IFN-γ and IL-2 secretion (**Figures 2D, E**). In long term antigen exposure functional assays, 80BB demonstrated sustained killing which was comparable with BB^ΔBRM^ (**Figure 2F**). However, it increased cytokine release (**Figure 2G**) and showed a trend of greater proliferation (**Figure 2H**). These advantages were recapitulated using the MOR scFv (see **Figures 3B, C**). Thus, 80BB provides stronger co-stimulation than 4-1BB^ΔBRM^ in the TCR-mimetic CAR platform.

**Figure 2:**
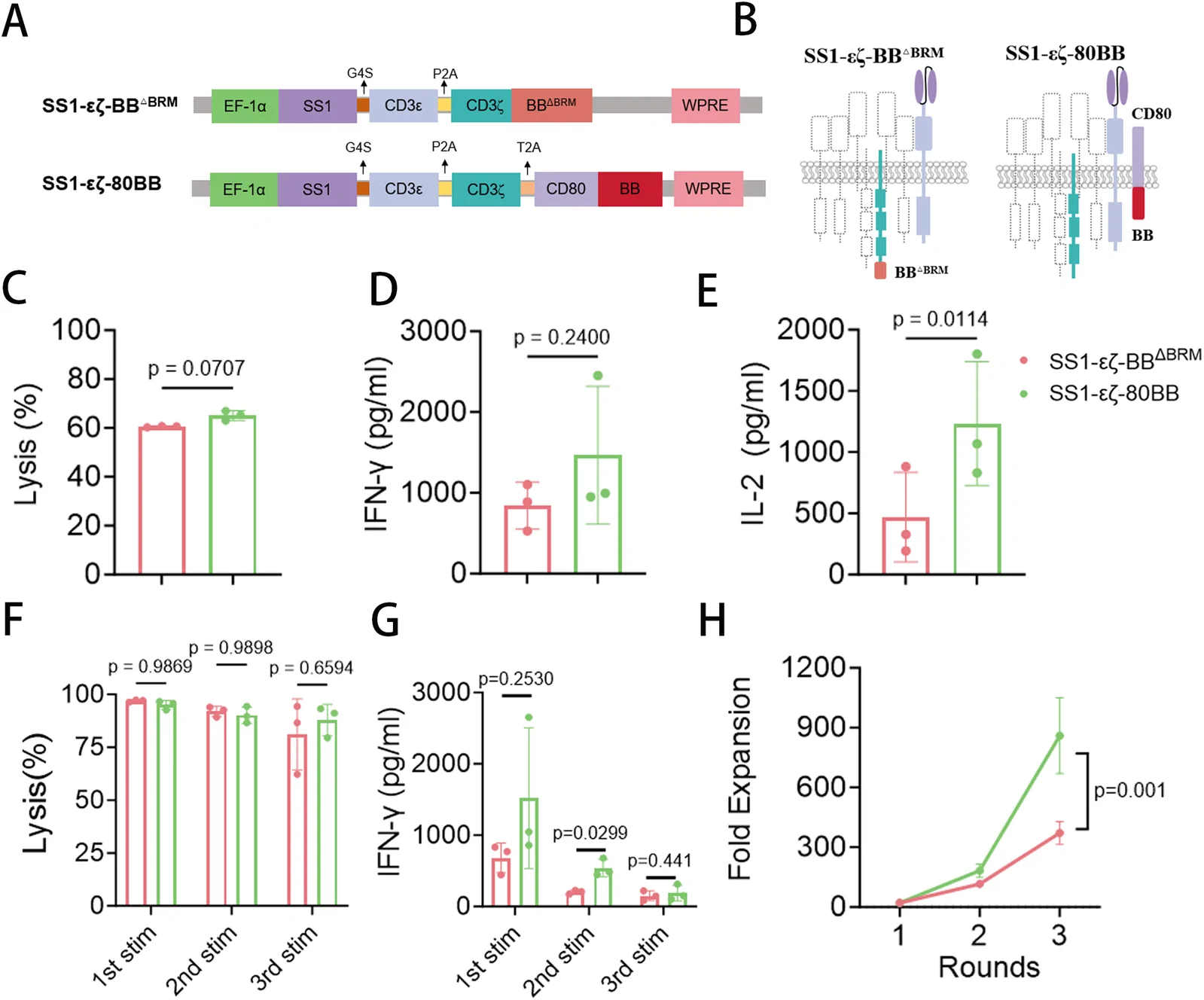
80BB co-stimulation is superior to 4-1BB^ΔBRM^ in TCR-mimetic CAR-T cells. **(A)** Schematic diagram of lentiviral vectors: SS1-ϵζ-BB^ΔBRM^ and SS1-ϵζ-80BB (SS1-ϵζ containing chimeric co-stimulation receptor that is CD80 ecto and transmembrane domain fused to 4-1BB ICD). **(B)** Schematic representation of SS1-ϵζ-BB^ΔBRM^ and SS1-ϵζ-80BB constructs within the TCR complex. **(C)** Cytotoxicity of SS1-ϵζ-80BB and SS1-ϵζ-BB^ΔBRM^ TCR-mimetic CAR-T cells against MSLN^+^ SKOV3-luc cells at an E:T ratio of 1:1. Data are from three independent donors, each shown as a single data point after averaging technical replicates (n = 3). *p* values were calculated by paired t-test. **(D, E)** IFN-γ **(D)** and IL-2 **(E)** concentrations in the supernatant 24 h after co-culture with MSLN^+^ SKOV3-luc target cells at an E:T ratio of 1:1. Data are from three independent donors, each shown as a single data point after averaging technical replicates (n = 3). *p* values were calculated by paired t-test. **(F, G)** Serial killing assay: Cytotoxic activity after each stimulation cycle **(F)** and IFN-γ production **(G)**. Data are from three independent donors, each shown as a single data point after averaging technical replicates (n = 3). *p* values were calculated by paired t-test at each cycle. **(H)** Antigen-based CAR-T expansion assay: Cumulative fold expansion of CAR-T cells over time following antigen expansion. Data are from three experimental replicates from one representative donor. Two-way repeated-measures ANOVA was performed for descriptive purposes; the data should be interpreted as exploratory. Data have been presented as mean ± SD.

**Figure 3:**
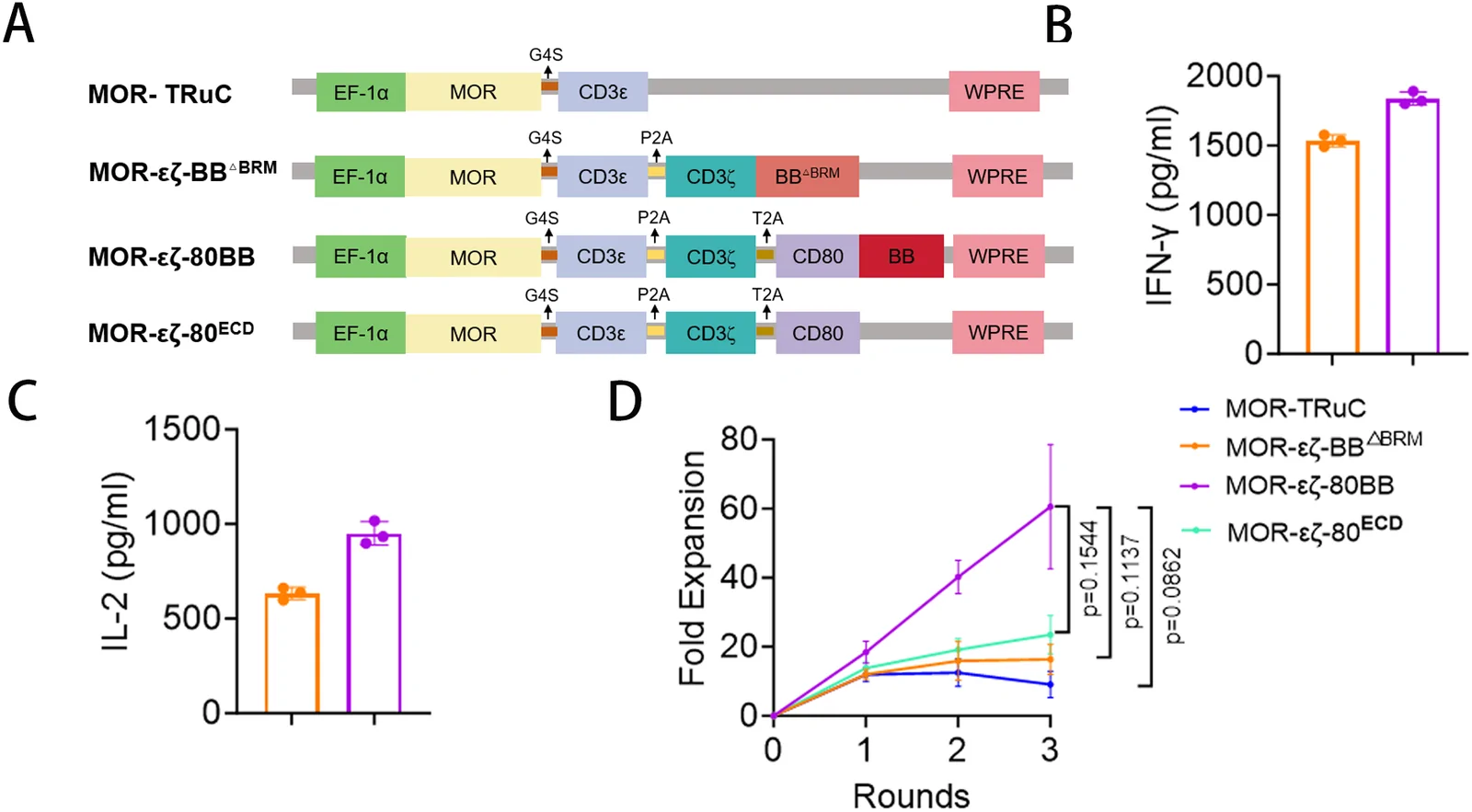
Lower-affinity MOR TCR-mimetic CAR-T recapitulate 80BB superiority. **(A)** Schematic diagram of low-affinity MOR antibody-based CAR variants: MOR-TRuC, MOR-ϵζ-BB^ΔBRM^, MOR-ϵζ-80BB, MOR-ϵζ-80^ECD^. **(B, C)** IFN-γ **(B)** and IL-2 **(C)** concentrations in the supernatant 24 h after co-culture with SKOV3 cells at an E:T ratio of 3:1. Data are from three technical replicates from one representative donor; no statistical inference was performed due to the absence of donor replicates. **(D)** Antigen-based CAR-T expansion assay: cumulative fold expansion of MOR-based CAR-T cells over time. Data are from three experimental replicates from the same donor (mean ± SD). Two-way repeated-measures ANOVA was performed for descriptive purposes; the data should be interpreted as exploratory.

### CD3ζ overexpression further optimizes 80BB-engineered TCR-mimetic CAR-T

After identifying 80BB as the optimal co-stimulatory module, we expressed CD3γ, CD3δ, or CD3ζ respectively alongside the ϵ-80BB backbone (lacking exogenous CD3ζ; **Figures 4A, B**) to determine whether or which CD3 subunits contribute to TCR-mimetic CAR-T function. Using two independent donors, we observed that only CD3ζ overexpression increased TCR-mimetic CAR surface MFI. Among CD3γ, CD3δ, and CD3ζ, the ζ subunit demonstrated a consistent trend toward the highest CAR surface expression (**Figure 4C**). We further compared SS1-ϵ (SS1-TRuC, without exogenous CD3ζ) with SS1-ϵζ (with exogenous CD3ζ), and SS1-ϵζ showed significantly higher surface CAR MFI than SS1-ϵ in three independent donors (**Figures 4F–H**). Moreover, SS1-ϵ consistently showed significantly lower CAR MFI than SS1-ϵζ-BB^ΔBRM^ and SS1-ϵζ-80BB in independent donors (**Figures 4F, G**).

**Figure 4:**
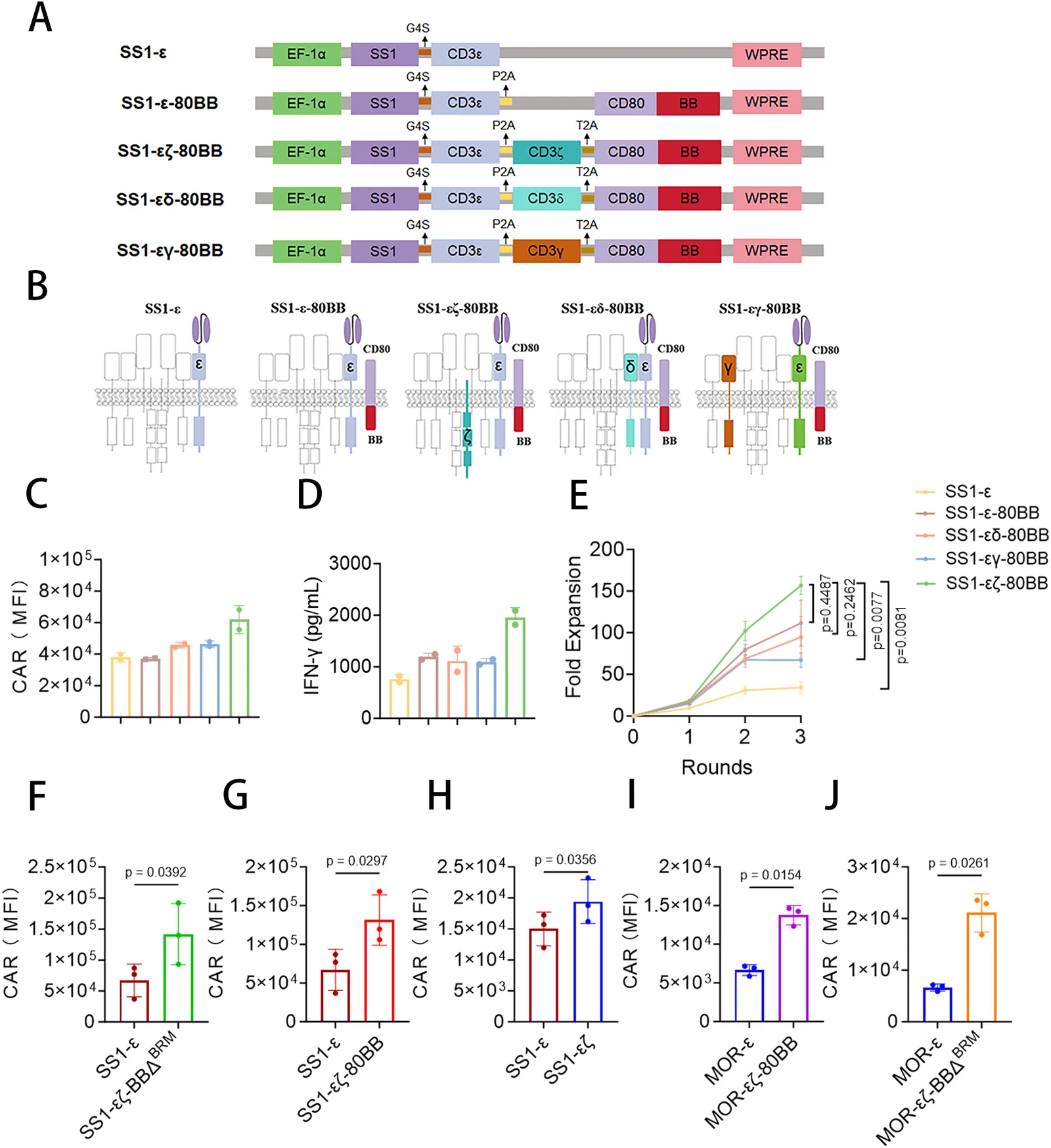
CD3ζ overexpression further optimizes 80BB-engineered TCR-mimetic CAR-Ts. **(A)** Schematic diagram of lentiviral vectors: SS1-ϵ, SS1-ϵ-80BB, SS1-ϵζ-80BB, SS1-ϵδ-80BB, and SS1-ϵγ-80BB. **(B)** Schematic representation of CAR within the TCR complex. **(C)** Data are from two independent donors (n = 2). Data are shown as descriptive trends; no statistical inference was performed. **(D)** Data are from two independent donors, each shown as a single data point after averaging technical replicates (n = 2). Data are shown as descriptive trends; no statistical inference was performed. **(E)** Antigen-based CAR-T expansion assay: Cumulative fold expansion of CAR-T cells over time following antigen stimulation. Data are from three experimental replicates from one representative donor. Two-way repeated-measures ANOVA was performed for descriptive purposes; the data should be interpreted as exploratory. Data are presented as mean ± SD. **(F)** Surface CAR MFI between SS1-ϵ and SS1-ϵζ-BB^ΔBRM^ measured by flow cytometry. Data are from three independent donors (n = 3). *p* values were calculated by two-tailed paired t-test. Data are presented as mean ± SD. **(G)** Surface CAR MFI between SS1-ϵ and SS1-ϵζ-80BB measured by flow cytometry. Data are from three independent donors (n = 3). *p* values were calculated by two-tailed paired t-test. Data are presented as mean ± SD. **(H)** Surface CAR MFI between SS1-ϵ and SS1-ϵζ measured by flow cytometry. Data are from three independent donors (n = 3). *p* values were calculated by two-tailed paired t-test. Data are presented as mean ± SD. **(I)** Surface CAR MFI between MOR-ϵ and MOR-ϵζ-80BB measured by flow cytometry. Data are from three independent donors (n = 3). *p* values were calculated by two-tailed paired t-test. Data are presented as mean ± SD. **(J)** Surface CAR MFI between MOR-ϵ and MOR-ϵζ-BB^ΔBRM^ measured by flow cytometry. Data are from three independent donors (n = 3). *p* values were calculated by two-tailed paired t-test. Data are presented as mean ± SD.

In short-term cytotoxicity analysis, SS1-ϵζ-80BB was comparable to SS1-ϵ-80BB (**Supplementary Figure S2C**). However, in two independent donors, CD3ζ-overexpressing CAR-T cells demonstrated a trend toward higher IFN-γ secretion upon short-term stimulation. (**Figure 4D**). In long term antigen exposure assays, they showed a trend of superior expansion in a representative donor (**Figure 4E**). In separate experiments using three independent donors, SS1-ϵζ also showed significantly higher IFN-γ and IL-2 secretion than SS1-ϵ (**Supplementary Figures S2E, F**). Therefore, CD3ζ overexpression was incorporated into the final design.

To assess whether this effect is scFv-independent, we also examined the MOR scFv. Consistently, MOR-ϵζ-BB^ΔBRM^ and MOR-ϵζ-80BB showed significantly higher CAR MFI than MOR-ϵ (without exogenous CD3ζ) across multiple donors (**Figures 4I, J**), confirming that the CD3ζ-mediated enhancement of TCR-mimetic CAR-T surface expression which is not dependent on the specific scFv.

### SS1-ϵζ-80BB TCR-mimetic CAR generates potent anti-tumor activity but *in vivo* lethal toxicity

In SKOV3 ovarian cancer xenografts, compared with SS1-ϵζ-BB^ΔBRM^, SS1-BBζ, SS1-TRuC, SS1-ϵζ-80BB (1×10^6^ cells per mouse) demonstrated superior tumor control (**Figures 5A, B**; **Supplementary Figure S3A**). SS1-ϵζ-80BB CAR-T cells exhibited high persistence in peripheral blood which was detectable at both day 30 and day 35 (**Figure 5D**; **Supplementary Figure S4A**). qPCR also confirmed higher CAR DNA copies in SS1-ϵζ-80BB peripheral blood samples from three mice per group (**Figure 5E**; **Supplementary Figure S3B**). At day 35, tumor-infiltrating human CD45^+^ cells were assessed in pooled tumor samples from two mice per group (**Supplementary Figure S4B**). An exploratory analysis showed a trend toward higher infiltration in the SS1-ϵζ-80BB group compared with the other groups. However, SS1-ϵζ-80BB was associated with unexpected lethality in mice that occurred early despite a minimal tumor burden, suggesting a systematic safety evaluation is required (**Figure 5C**).

**Figure 5:**
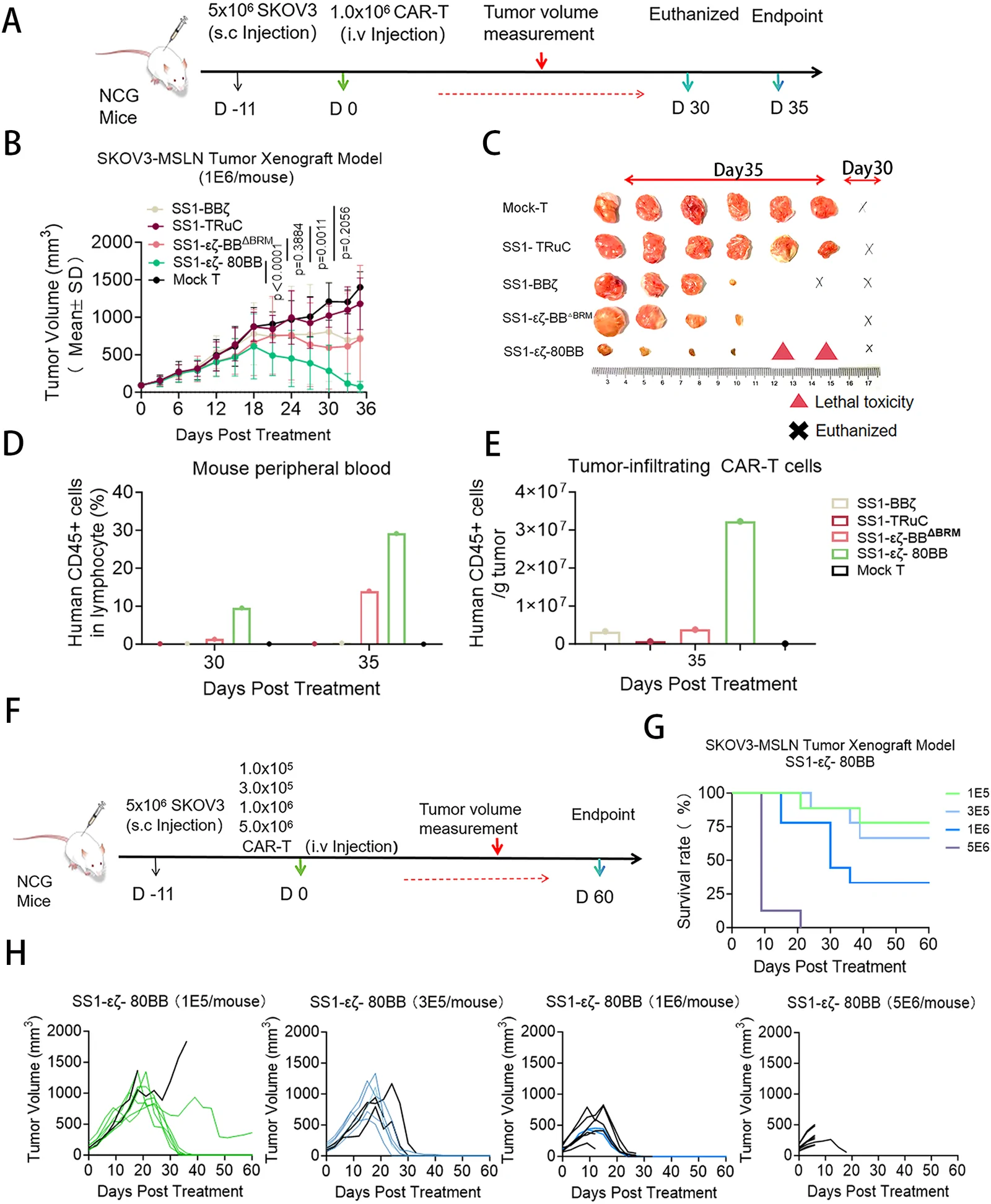
SS1-ϵζ-80BB TCR-mimetic CAR-T generate potent anti-tumor activity but *in vivo* lethal toxicity. **(A)** Experimental roadmap: NCG mice received 5×10^6^ SKOV3 cells (right flank, day 0) and were randomized on day 11 for a single injection of 1×10^6^ CAR-T cells (n = 7 mice per group). **(B)**Tumor growth was measured with a caliper. *p* values were calculated by mixed-effects models with group, time, and group×time interaction as fixed effects, and individual mouse as a random effect. Data are presented as mean ± SD. **(C)** Representative excised tumors from each group at endpoint (day 35). **(D)** Quantified percentages of peripheral blood CD45^+^ cells by flow cytometry(day 30 and 35), this is an exploratory analysis and no statistical inference was performed. **(E)** Quantification of tumor-infiltrating human CD45^+^ CAR-T cells by counting beads assay on day 35. CAR-T cells were pooled from tumors of two mice per experimental group for analysis, this is an exploratory analysis and no statistical inference was performed. **(F)** Dose-dependent toxicity: NCG mice bearing SKOV3 tumors received a single intravenous injection of SS1-ϵζ-80BB TCR-mimetic CAR-T cells (1×10^5^, 3×10^5^, 1×10^6^, or 5×10^6^ CAR^+^ cells; n = 8 mice per group). **(G)** Survival curves for the different dose cohorts (log-rank Mantel–Cox test). **(H)** Tumor growth curves for the different dose cohorts. Bold black lines indicate mice that succumbed.

### SS1-ϵζ-80BB TCR-mimetic CAR displays dose-dependent toxicity

Dose-escalation studies (1×10^5^, 3×10^5^, 1×10^6^, 5×10^6^ CAR-T cells per mouse) revealed clear dose-dependent toxicity (**Figure 5F**). At 5×10^6^ cells, 7/8 mice died within 7 days; at 1×10^6^, 2/8 died within 18 days; at 3×10^5^, 3/8 died despite tumor clearance at day 39; at 1×10^5^, only 2/8 died from tumor progression (**Figures 5G, H**).

Off-target recognition was assessed by membrane proteome array (MPA) (**Supplementary Figures S6A–C**) and surface plasmon resonance (SPR). The MPA identified MSLN as the expected cell membrane target and TMEM43 as an intracellular target which was not independently validated in the present study. No functional off-target risk was detected under the assay conditions. The SS1 scFv binds human MSLN (KD = 3.08×10^−10^ M), but not murine MSLN (**Supplementary Figures S6D, E**). An *in vitro* IL-6 production assay showed a trend of higher IL-6 secretion by SS1-ϵζ-80BB than by other CAR constructs tested in two donors (**Supplementary Figure S3C**). These data indicate that toxicity is associated with T cell activation rather than off-target binding.

### Deletion of the 4-1BB intracellular domain from 80BB abolish *in vivo* toxicity

To retain CD80-mediated inter-cellular co-stimulation, we ablated 4-1BB ICD (intracellular domain) of SS1-ϵζ-80BB and generated SS1-ϵζ-80^ECD^ (**Figures 6A, B**). Surface CD80 expression was comparable between 80BB and 80^ECD^ (**Supplementary Figure S7A**), confirming that deletion of the 4-1BB intracellular domain did not affect CD80 ectodomain membrane expression. Furthermore, to test whether 4-1BB signaling delivered via a different mode could restore toxicity, we constructed SS1-ϵζ-BB^ΔBRM^-80^ECD^, which combines BB^ΔBRM^ (fused to CD3ζ) instead of 80 fusion.

**Figure 6:**
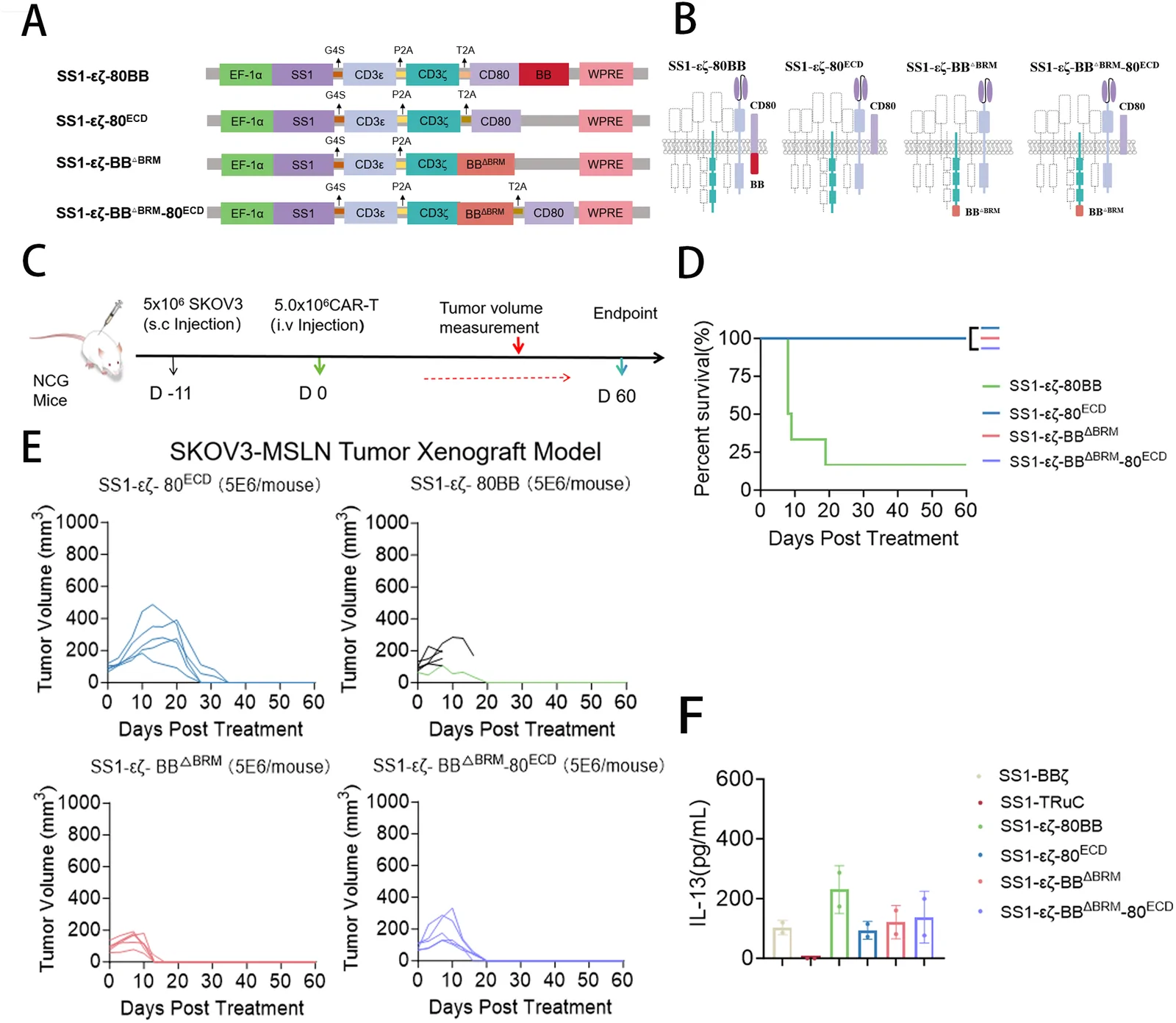
Deletion of the 4-1BB intracellular domain from 80BB abolishes *in vivo* toxicity. **(A)** Schematic diagram of lentiviral vectors: SS1-ϵζ-BB^ΔBRM^, SS1-ϵζ-80BB, SS1-ϵζ-80^ECD^ (CD80 ectodomain retained; entire 4-1BB ICD deleted) and SS1-ϵζ-BB^ΔBRM^-80^ECD^. **(B)** Schematic representation of CAR constructs within the TCR-CD3 complex. **(C)** NCG mice bearing SKOV3 tumors (5×10^6^ cells, right flank, day 0) were randomized on day 11 to receive a single intravenous injection of 5×10^6^ CAR-T cells of either SS1-ϵζ-BB^ΔBRM^, SS1-ϵζ-80BB, SS1-ϵζ-80^ECD^, or SS1-ϵζ-BB^ΔBRM^-80^ECD^ (n = 5–6 mice). **(D)** Survival curves (log-rank Mantel–Cox test). **(E)** Tumor growth curves; bold black lines indicate mice that succumbed. **(F)** IL-13 concentrations in the supernatant 24 h after co-culture with MSLN^+^ SKOV3 cells at an E:T ratio of 1:1. Data are from two independent donors, each shown as a single data point after averaging technical replicates (n = 2). Data are shown as descriptive trends; no statistical inference was performed.

Both SS1-ϵζ-80ECD and SS1-ϵζ-BB^ΔBRM^-80^ECD^ demonstrated an excellent safety profile; all mice achieved 100% survival beyond 60 days alongside complete tumor regression (**Figures 6C–E**). Similarly, SS1-ϵζ-BB^ΔBRM^ exhibited comparable safety. In contrast, 5/6 mice died in the SS1-ϵζ-80BB cohort within 20 days (**Figures 6D, E**).

Thus, the 4-1BB intracellular domain caused the lethality, but only when it was part of the intact 80BB chimera—not when it was attached to CD3ζ as BB^ΔBRM^. Consistently, IL-13 (a downstream effector of 4-1BB) (30), showed a trend of higher expression in 80BB-containing constructs compared with those lacking the 4-1BB intracellular domain in two donors (**Figure 6F**). IL-13 levels were reduced upon 4-1BB ICD deletion, with comparably low levels observed in SS1-ϵζ-BB^ΔBRM^, SS1-ϵζ-BB^ΔBRM^-80^ECD^ and SS1-BBζ, and it was nearly undetectable in SS1-TRuC TCR-mimetic CAR-T (**Figure 6F**).

### MOR-based TCR-mimetic CAR-T recapitulate 80BB superiority

To determine whether the observed SS1-specific phenotype was dependent on the scFv, we generated MOR-based constructs with the same architectures as their SS1 counterparts: MOR-TRuC, MOR-ϵζ-BB^ΔBRM^, MOR-ϵζ-80BB, and MOR-ϵζ-80ECD (**Figure 3A**). MOR binds human MSLN with a KD of 2.2×10⁻^9^ M, approximately sevenfold lower affinity than SS1 (KD = 3.08×10⁻^10^ M), and cross-reacts with murine MSLN (**Supplementary Figures S8A, B**).

In a representative donor, MOR-ϵζ-80BB showed a trend of higher cytokine secretion (IFN-γ and IL-2) (**Figures 3B, C**) and expansion (**Figure 3D**) compared with MOR-ϵζ-BB^ΔBRM^, consistent with SS1 results. MOR-ϵζ-80BB TCR-mimetic CAR-T cells showed a trend toward the strongest expansion among the constructs tested in this representative donor. Deletion of the 4-1BB intracellular domain (MOR-ϵζ-80^ECD^) reduced proliferation, and MOR-TRuC (lacking any co-stimulatory module) showed the weakest expansion (**Figure 3D**).

### Comparison of SS1- and MOR-based 80BB TCR-mimetic CAR-T cells *in vitro* and *in vivo*

We compared the function of SS1-ϵζ-80BB and MOR-ϵζ-80BB TCR-mimetic CAR-T cells *in vitro* using the same CAR-T cell product that were subsequently used for the *in vivo* experiment (**Supplementary Figure S9A**). In one representative donor, upon antigen stimulation, MOR-ϵζ-80BB CAR-T cells secreted lower levels of IFN-γ (**Supplementary Figure S9B**) and IL-2 (**Supplementary Figure S9C**) than SS1-ϵζ-80BB CAR-T cells. In addition, SS1-ϵζ-80BB CAR-T cells showed higher IL-6 secretion (**Supplementary Figure S9D**) and greater expansion capacity upon repeated stimulation (**Figure 7B**). By contrast, the MOR-ϵζ-80BB construct showed substantially weaker expansion under the same conditions.

**Figure 7:**
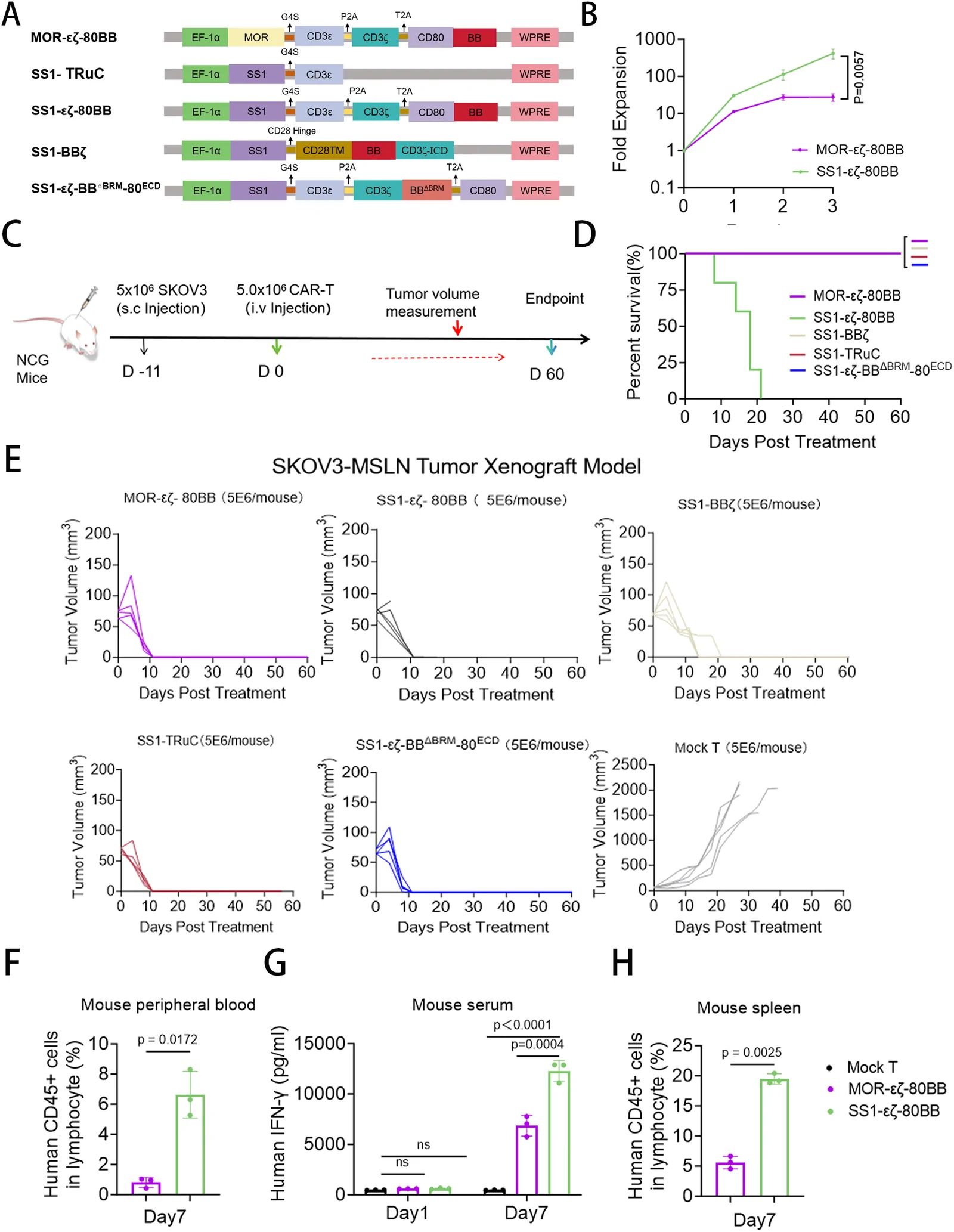
Comparison of SS1- and MOR-based 80BB TCR-mimetic CAR-T cells *in vitro* and *in vivo*. **(A)** Schematic diagram of lentiviral vectors: MOR-ϵζ-80BB, SS1-ϵζ-80BB, SS1-ϵζ-BB^ΔBRM^-80^ECD^, SS1-TRuC, and SS1-BBζ (conventional 4-1BBζ CAR). **(B)** Antigen-based CAR-T expansion assay: Cumulative fold expansion of MOR-ϵζ-80BB and SS1-ϵζ-80BB CAR-T cells over time following antigen stimulation. Data are from three experimental replicates from one representative donor (mean ± SD). Two-way repeated-measures ANOVA was performed for descriptive purposes; the data should be interpreted as exploratory. **(C)** NCG mice were engrafted subcutaneously with SKOV3 cells (5×10^6^ cells, right flank, day 0) and on day 11 received 5×10^6^ CAR-T cells: MOR-ϵζ-80BB, SS1-ϵζ-80BB, SS1-TRuC, or SS1-BBζ (n = 5 mice per group for tumor growth and survival monitoring; an additional three satellite mice in the MOR-ϵζ-80BB and SS1-ϵζ-80BB groups were used for monitoring pathological/histological changes). **(D)** Survival curves (log-rank Mantel–Cox test). **(E)** Tumor growth curves; bold black lines indicate mice that succumbed. **(F)** Percentage of human CD45^+^ cells in peripheral blood of MOR-ϵζ-80BB and SS1-ϵζ-80BB satellite mice on day 7, when SS1-ϵζ-80BB showed lethal toxicity. Data are from three biologically independent mice (n = 3). *p* values were calculated by two-tailed unpaired t-test. **(G)** Serum IFN-γ concentrations of MOR-ϵζ-80BB and SS1-ϵζ-80BB satellite mice, and in the mock-transduced T group on day 1 (18 h post-infusion) and day 7. Data are from three biologically independent mice (n = 3). *p* values were calculated by one-way ANOVA. **(H)** Percentage of human CD45^+^ cells in the spleen of MOR-ϵζ-80BB and SS1-ϵζ-80BB satellite mice on day 7. Data are from three biologically independent mice (n = 3). *p* values were calculated by two-tailed unpaired t-test. Data have been presented as mean ± SD.

To compare SS1-ϵζ-80BB and MOR-ϵζ-80BB TCR-mimetic CAR-T *in vivo*, we administered a high dose (5×10^6^ cells per mouse) of the indicated CAR-T cell types using a second, independent donor (**Figures 7A, C**). SS1-BBζ, SS1-TRuC and SS1-ϵζ-BB^ΔBRM^-80^ECD^ were included as controls. All mice in the SS1-ϵζ-80BB group died within 20 days, whereas no mortality occurred in the MOR-ϵζ-80BB or any control group (**Figure 7D**). This indicates that the SS1 scFv itself was not sufficient to induce lethal toxicity when paired with conventional CAR (SS1-BBζ) or TRuC (SS1-TRuC) architectures. The safety of SS1-ϵζ-BB^ΔBRM^-80^ECD^ was reproduced in this second donor, whereas the lethal phenotype of SS1-ϵζ-80BB was again observed(**Figures 7D, E**). Mock T-treated mice eventually succumbed to tumor progression when tumor volumes reached the humane endpoint.

Notably, the first deaths in the SS1-ϵζ-80BB cohort occurred on day 7. At this time point, we euthanized three satellite mice from each group (SS1-ϵζ-80BB and MOR-ϵζ-80BB) and also collected tissues from mice that succumbed early with minimal tumor burden. Histological analysis was performed on liver, spleen, and lung sections using H&E staining, and human CD3 IHC was performed on liver and lung. In parallel, CAR-T expansion in peripheral blood was assessed by flow cytometry, and splenic CAR-T proportion, phenotype, and CD4/CD8 ratios were also analyzed.

Blood analysis revealed greater peripheral CAR-T expansion in the SS1-ϵζ-80BB group than in the MOR-ϵζ-80BB group (**Figure 7F**; **Supplementary Figure S10A**). Serum cytokine analysis detected elevated human IFN-γ levels in the SS1-ϵζ-80BB group (**Figure 7G**). Flow cytometry further confirmed a higher CAR-T proportion in the spleen of SS1-ϵζ-80BB mice compared to MOR-ϵζ-80BB mice (**Figure 7H**; **Supplementary Figures S10B, C**). The two groups showed no significant differences in CD4/CD8 ratios (**Supplementary Figure S10D**) or phenotype (defined as Tcm, Tem, Tn/Tscm, Teff) (**Supplementary Figure S10E**).

IHC quantification was performed using HALO whole-slide analysis, which showed human T cell infiltration in the lungs and liver of SS1-ϵζ-80BB mice. In contrast, infiltration was substantially lower in MOR-ϵζ-80BB mice (**Supplementary Figures S11A, B**). H&E staining evaluated by blinded histopathological scoring showed inflammatory injury and structural disruption in the liver and lung of SS1-ϵζ-80BB mice, with organ damage observed in mice that died early with minimal tumor burden, whereas MOR-ϵζ-80BB mice showed substantially milder changes (**Supplementary Figures S12**, **S13**). Together, these findings were consistent with the treatment-associated lethality observed in the SS1-ϵζ-80BB group.

## Discussion

The prevailing paradigm in CAR-T optimization posits that stronger signaling enhances antitumor efficacy. We herein report a paradoxical finding: stepwise optimization of costimulation and CAR density in TCR-mimetic CAR-T cells achieved potent activity but at the cost of lethal toxicity. This outcome reframes the central challenge—shifting the focus from maximizing potency to deciphering how affinity and costimulatory architecture govern safety in this platform.

Our optimization strategy builds upon and extends two pivotal prior studies. Lah et al. (21) showed that the BRM motif of 4-1BB impairs TCR complex assembly when 4-1BB is fused directly to CD3ζ, and we recapitulate their finding that BRM deletion enhances *in vitro* function. Dobrin et al. (8) demonstrated that 80BB improves efficacy without toxicity in TCR-T and HIT platforms. We extend this work by showing that, in TCR-mimetic CAR-T cells, 80BB outperforms 4-1BB^ΔBRM^ across two scFvs. Critically, however, we observed a scFv-dependent safety divergence: the SS1-based construct induced toxicity, whereas the MOR-based construct did not under identical experimental conditions, revealing that the safety profile of the 80BB architecture is inherently linked to the scFv employed.

We further observed that CD3ζ overexpression consistently increased surface TCR-mimetic CAR levels across both SS1 and MOR scFvs in multiple donors—an effect not recapitulated by CD3γ or CD3δ overexpression. Given that the TCR complex comprises ϵδ and ϵγ heterodimers paired with a ζζ homodimer (31), and that Degirmenci et al. reported a T-cell expression hierarchy of γ > δ > ϵ > ζ (29), while CD3ζ downregulation is known to impair T-cell function (28), these surface expression data suggest that CD3ζ co-expression augments CAR surface density. We note, however, that direct biochemical validation—including Western blot, co-immunoprecipitation, or blue-native PAGE—was not performed here and remains an important direction for future work.

The fully optimized SS1-ϵζ-80BB design induced dose-dependent lethal toxicity. Off-target binding was systematically assessed by membrane proteome array and surface plasmon resonance. The MPA identified MSLN as the expected target and also detected a signal for TMEM43, a known intracellular protein; no functional off-target risk was detected under the assay conditions. Mortality was observed exclusively in mice receiving the intact 80BB chimera; all control groups—including SS1-TRuC, SS1-BBζ, SS1-ϵζ-80^ECD^, and SS1-ϵζ-BB^ΔBRM^-80^ECD^—survived, ruling out architecture-intrinsic toxicity, SS1 scFv-specific effects, CD80 ectodomain-driven effects, and the combination of CD80 engagement with antigen-dependent 4-1BB signaling (**Figure 8**). Moreover, the prolonged survival (>27 days) of mice receiving high-dose untransduced T cells, together with the absence of mortality in all control CAR-T groups despite equivalent CAR^+^ cell doses, excludes xenogeneic graft-versus-host disease (GVHD) as an alternative explanation. *In vitro* IL-6 production was elevated in the SS1-ϵζ-80BB group, raising the possibility that such signals, in the presence of myeloid cells, could contribute to cytokine-related toxicity in the clinical setting (32–36).

**Figure 8:**
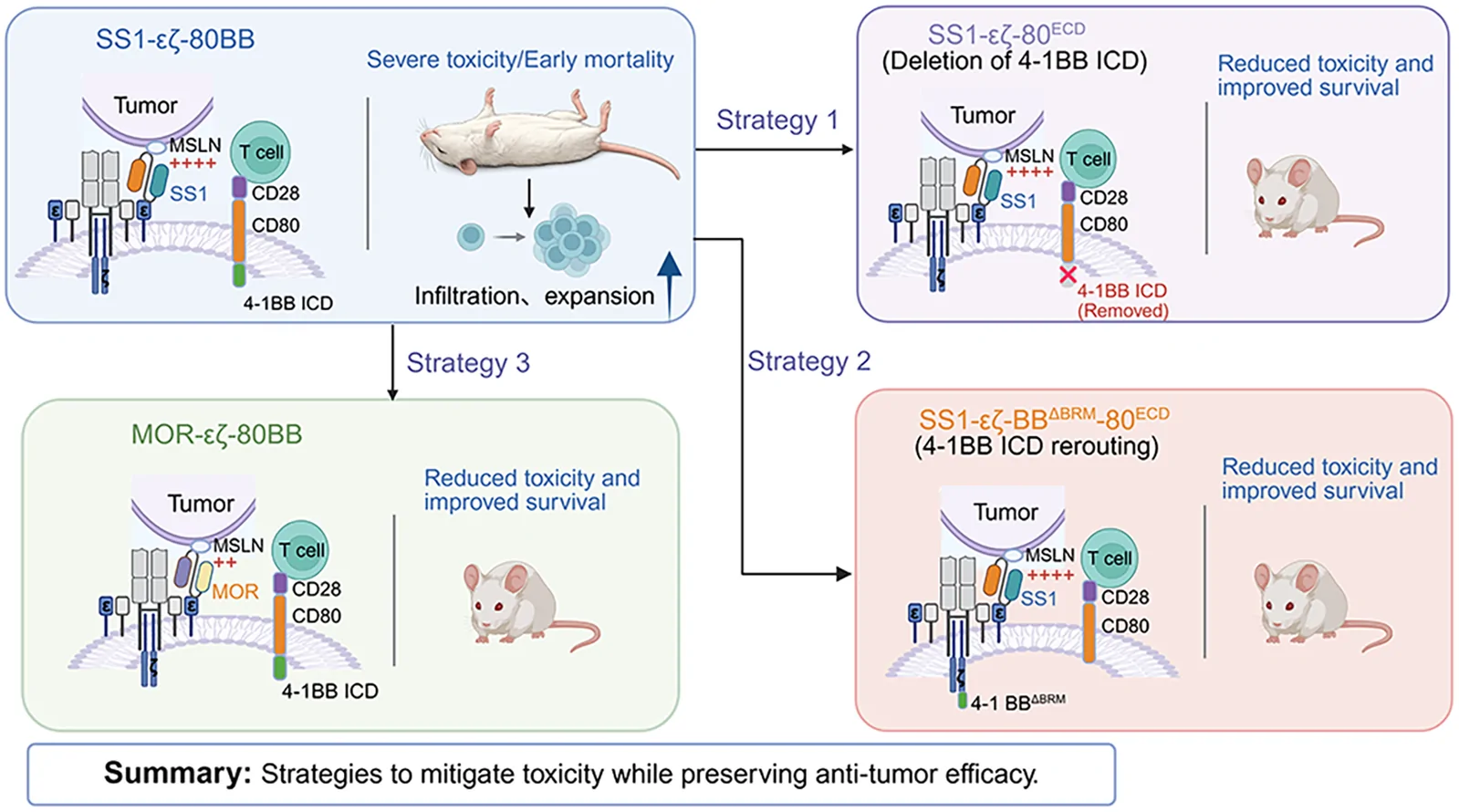
Strategies to mitigate toxicity while preserving anti-tumor efficacy. Replacing SS1 with MOR, deleting the 4-1BB intracellular domain (80^ECD^), or rerouting the 4-1BB intracellular domain to an antigen-dependent format (BB^ΔBRM^-80^ECD^) each preserved efficacy while reducing toxicity in the mesothelin-targeted TCR-mimetic CAR-T platform.

SS1-ϵζ-80BB T cells exhibited substantially greater expansion than MOR-ϵζ-80BB T cells both *in vitro* and *in vivo*. IL-13 has been reported as a downstream effector of 4-1BB signaling (30). In our constructs, IL-13 secretion was elevated in 80BB-containing constructs and was reduced upon deletion of the 4-1BB intracellular domain. Histological analysis, including HALO-based whole-slide quantification, demonstrated massive human CD3^+^ T-cell infiltration in the liver and lungs specifically in the SS1-ϵζ-80BB group, whereas infiltration was substantially lower in the MOR-ϵζ-80BB group (**Supplementary Figures S11A, B**), consistent with the association between lethal toxicity and extensive T-cell infiltration into these organs (37). H&E staining evaluated by blinded histopathological scoring revealed inflammatory injury and structural disruption in the liver and lung of SS1-ϵζ-80BB mice, with organ damage observed specifically in those that died early with minimal tumor burden, whereas MOR-ϵζ-80BB mice showed substantially milder changes (**Supplementary Figures S12**, **S13**).

The requirement for the intact 80BB chimera, as opposed to the 4-1BB intracellular domain alone, is supported by two independent observations. First, deletion of the 4-1BB intracellular domain from the 80BB chimera (80^ECD^) completely eliminated toxicity while preserving anti-tumor efficacy. Second, rerouting the same 4-1BB intracellular domain to an antigen-dependent, CD3ζ-linked format (BB^ΔBRM^-80^ECD^) was equally safe. Notably, delivery of the 4-1BB intracellular domain alone via BB^ΔBRM^ fused to CD3ζ was insufficient to cause death, indicating that the intact 80BB chimera is required to elicit the lethal phenotype. The safety of BB^ΔBRM^-80^ECD^, together with the lethality of 80BB, was reproduced in two independent donors. In parallel, replacing SS1 with the MOR scFv also abrogated toxicity without compromising efficacy in the tested system. We acknowledge that SS1 and MOR differ in epitope specificity, framework sequence, and other biophysical properties, and that they are not isogenic affinity variants derived from a single parental clone; therefore, the observed effect cannot be attributed solely to affinity, and this comparison does not constitute a systematic affinity titration. Together, these observations support the notion that attenuating either the 80BB-based architecture or the affinity-related properties preserves anti-tumor activity while reducing lethality.

Our findings offer potential design considerations for clinical CAR-T development. First, the comparison between SS1- and MOR-based constructs suggests that maximizing binding affinity may not be necessary for mesothelin-targeted platforms, thereby providing a useful reference point for scFv selection. Of note, this reference is specific to the mesothelin-targeted TCR-mimetic CAR-T platform and should not be extrapolated as a universal threshold applicable to other antigens or scFv families. Second, the mode of costimulatory configuration—whether the signal is delivered in an antigen-independent manner (80BB) or in an activation-dependent format (BB^ΔBRM^)—was associated with differential safety outcomes. Encouragingly, both strategies—80^ECD^ (deletion of the 4-1BB ICD) and BB^ΔBRM^-80^ECD^ (rerouting 4-1BB signaling to an antigen-dependent format)—effectively detoxified the 80BB platform without compromising its full therapeutic potency.

Our study supports and extends the work of Dobrin et al. (8), While they established 80BB as safe and efficacious in conventional TCR-T and HIT platforms, we observed that the SS1-based construct was associated with a narrower safety profile than the MOR-based construct in the TCR-mimetic CAR-T architecture. Importantly, SS1 was well tolerated in conventional BBζ CARs (38, 39) and in our SS1-BBζ and SS1-TRuC controls, further supporting the notion that co-stimulatory configuration influences the safety profile of a given scFv. Moreover, prior work has established that variations in antibody affinity may lead to distinct safety characteristics (40, 41).

This study has several limitations. First, our conclusions are based on a single antigen (mesothelin) and a single xenograft model (SKOV3); validation in additional antigens and tumor models is required to broaden generalizability. Second, although we provide functional and histological evidence for the proposed interpretation, we did not directly measure downstream phospho-signaling pathways (e.g., NF-κB, AKT, ERK), which have been characterized for 80BB in prior work (8), our mechanistic model is therefore based on functional readouts and indirect markers such as IL-13. Third, the *in vitro* IL-6 production assay lacks myeloid components and therefore does not fully recapitulate the complex cellular networks underlying clinical cytokine release syndrome. We also acknowledge that serum samples from the *in vivo* experiments were insufficient for comprehensive multiplex cytokine analysis beyond IFN-γ, and we did not perform complete blood counts or serum biochemical analyses for liver/kidney injury markers. Fourth, we did not perform systematic affinity titration using point-mutant variants derived from a single parental scFv; such studies would be valuable for more precisely defining the relationship between scFv properties and toxicity. We also acknowledge that we did not use calibrated beads to determine absolute MSLN molecules per cell on the target cell lines, and we did not test antigen-negative or MSLN-knockout targets, nor did we evaluate antigen-density variants spanning low, intermediate, and high MSLN expression levels. In addition, histopathological evaluation was primarily compared between SS1-ϵζ-80BB and MOR-ϵζ-80BB groups, and was not systematically assessed across all control groups (e.g., SS1-BBζ, SS1-TRuC, Mock T). Finally, we used FSC/SSC gating for dead-cell exclusion without a viability dye, and FMO controls were not performed for all markers. These limitations should be addressed in future studies.

In summary, our data suggest that the SS1-ϵζ-80BB architecture was associated with dose-dependent lethal toxicity in CD3ζ-enhanced TCR-mimetic CAR-T cells. Three independent engineering strategies—deleting the 4-1BB intracellular domain, rerouting it to an antigen-dependent format, or replacing SS1 with MOR—abrogated toxicity while preserving potent anti-tumor activity. These findings highlight that balancing scFv properties and co-stimulatory configuration may inform the safety profile of mesothelin-targeted TCR-mimetic CAR-T cells, offering potential design considerations for affinity selection and co-stimulator design in this platform.

## Materials and methods

### Cell lines

HEK-293T, SKOV3, and OVCAR3 cells were obtained from the American Type Culture Collection (ATCC). SKOV3-luc cells were then generated by transducing SKOV3 with a firefly luciferase-encoding lentivirus (Nanjing CART Medical Science and Technology Co., Ltd.), followed by puromycin selection to establish a stable pool. All cell lines were authenticated by STR profiling and were used within 10 passages after thawing and were regularly tested for mycoplasma contamination (negative throughout the study). All cell lines were maintained in DMEM or McCoy’s 5A medium supplemented with 10% heat-inactivated fetal bovine serum (FBS) at 37 °C in 5% CO_2_.

### Construction of CAR vectors

All CAR constructs were based on an MSLN-targeting scFv (SS1, US Patent 6809184; or MOR, WO 2009/068204 A1) fused to the N-terminus of CD3ϵ via a GGGGS linker. To evaluate antigen-dependent co-stimulation, three SS1-based constructs were generated. In SS1-ϵζ, the CD3ζ chain was linked to the C-terminus of CD3ϵ via P2A. In SS1-ϵζ-BB and SS1-ϵζ-BB^ΔBRM^, the full-length 4-1BB ICD and its BRM-truncated version were respectively fused in-frame to the C-terminus of CD3ζ. For antigen-independent co-stimulation, the 80BB chimera (CD80 ectodomain and transmembrane region fused to the 4-1BB ICD) was independently expressed via a T2A linker, creating SS1-ϵζ-80BB. To investigate the role of CD3 subunits, we constructed SS1-ϵ-80BB (without exogenous CD3ζ), SS1-ϵδ-80BB (with exogenous CD3δ), and SS1-ϵγ-80BB (with exogenous CD3γ). As comparators, we used SS1-BBζ, a conventional second-generation CAR comprising CD28 hinge/transmembrane and 4-1BB/CD3ζ signaling domains, and SS1-TRuC, an alternative TCR-based fusion construct in which the SS1 scFv is fused to CD3ϵ.

To evaluate the role of the 4-1BB ICD in 80BB-mediated toxicity, we generated SS1-ϵζ-80^ECD^ and SS1-ϵζ-BB^ΔBRM^-80^ECD^, in which the 4-1BB ICD was deleted from the 80BB module.

To test the effect of reduced antigen affinity, we replaced SS1 with a lower-affinity scFv, MOR. Following the same strategy, MOR-ϵ (MOR-TRuC), MOR-ϵζ-BB^ΔBRM^, MOR-ϵζ-80BB, and MOR-ϵζ-80^ECD^ were constructed. All constructs were verified by restriction digestion and Sanger sequencing before lentiviral packaging.

### Lentiviral production and T cell production

For lentiviral vector production, HEK293T cells at 70–80% confluence were co-transfected with CAR expression plasmids and packaging plasmids (pCMV-gag-pol, pVSV-G, pRSV-Rev) at a mass ratio of 1:1:1:1 (total DNA per T150 flask = 45 µg). Transfection was performed using polyethylenimine (PEI) at a DNA: PEI ratio of 1:3 (w/w). A complete medium change was carried out 4–6 h post-transfection. At 24 h and 48 h post-transfection, the supernatant was collected, filtered through a 0.45 µm membrane, ultracentrifuged (24,000×g, 2–3 h), resuspended in Opti-MEM, and stored at −80 °C.

For CAR-T cell production, PBMCs were obtained from healthy donors under written informed consent. CD3^+^ T cells were isolated from healthy donor peripheral blood mononuclear cells (PBMCs) using the EasySep™ Human T Cell Isolation Kit and activated with Dynabeads™ Human T-Activator CD3/CD28 at a bead-to-cell ratio of 3:1. Cells were cultured in OpTmizer™ CTS™ Medium supplemented with 100 U/mL IL-2, 1% L-glutamine, and 5% FBS. Lentiviral transduction was performed 24 h post-stimulation, and the cell density was maintained at 0.5–2×10^6^ cells/mL during expansion. After bead removal, CAR expression was verified by flow cytometry, and the cells were then frozen and stored for subsequent functional assays.

### Flow cytometry analysis *in vitro*

For cell surface marker analysis, cells were washed with phosphate-buffered saline (PBS; Cytiva) containing 5% FBS (Excell) and then stained with fluorochrome-conjugated antibodies for 30 min. After two additional washes, samples were acquired on an ACEA Novocyte 3110 flow cytometer. The flow cytometer used in this study is a 3-laser, 13-color system (488/640/405 nm). The antibody combinations used in this study had minimal spectral overlap. Dead cells and debris were excluded based on FSC/SSC gating. Acquisition settings were standardized across batches using identical PMT voltages, and gating was applied using a consistent prospective template. A minimum of 20,000 events were acquired per sample for analyses. FSC-A vs FSC-H gating for doublet exclusion was applied in some experiments (e.g., **Supplementary Figures S1**, **S7**) but was not uniformly applied across all experiments. We acknowledge the lack of uniform singlet discrimination as a limitation.

Mesothelin (MSLN) expression was assessed by PE-conjugated anti-MSLN antibody (Abcam, ab252136) staining. SS1-CAR expression was detected by sequential staining with biotinylated anti-mouse IgG F(ab’)_2_ fragment (Jackson ImmunoResearch, 115-065-072) followed by PE-streptavidin (BioLegend, 405203). MOR-CAR expression was detected by biotinylated anti-human IgG F(ab’)_2_ fragment (Jackson ImmunoResearch, 109-066-006) followed by PE-streptavidin. 80BB chimera expression was analyzed with APC-anti-CD80 (BioLegend, 375404). For antigen-stimulated expansion assays, cell numbers were quantified using Precision Count Beads™ (BioLegend, 424902).

### Cytotoxicity and functional persistence assay

Cytolytic activity was assessed by co-culturing SKOV3-Luc target cells (2×10^4^ cells/well) with mock-transduced (control) or CAR-T cells at various effector-to-target (E:T) ratios in IL-2-free medium for 24 h. After incubation, 100 µL of supernatant was collected for cytokine analysis, and an equal volume of One-Glo™ luciferase assay reagent (Promega, E6120) was added to the remaining culture. Following a 10-min incubation, luminescence was measured using a Fluoroskan Ascent FL microplate reader (Thermo Scientific). Specific cytotoxicity was calculated as [(control luminescence - experimental luminescence)/control luminescence] × 100%.

To assess multi-cycle cytotoxicity, SKOV3-luc target cells and CAR-T cells were co-cultured on day 0 at an effector-to-target (E:T) ratio of 2:1 in OpTmizer™ medium (5% FBS, 1% GlutaMAX™) without IL-2. After 48 h, CD3^+^ T cells were harvested, resuspended in 1 mL, and split into 300 µL for cytotoxicity assessment and 700 µL for restimulation (co-cultured with 5×10^5^ fresh target cells). This cycle was repeated every 48 h for three rounds. Serial killing was evaluated by specific lysis, and functional persistence was assessed by IFN-γ production.

To assess antigen-stimulated expansion capacity, SKOV3 target cells (1×10^6^) were seeded in T25 flasks 24 h prior to co-culture (day-1). On day 0, CAR-T cells were added at an effector-to-target (E:T) ratio of 0.5:1 (5×10^5^ CAR-T cells per flask) in OpTmizer™ medium. CAR-T cells were harvested, counted, and then re-plated with freshly seeded target cells at the same E:T ratio. On day 6 of each cycle, absolute CD3^+^ T-cell counts were determined by flow cytometry using Precision Count Beads™ (or a CountStar automated cell counter), and the fold expansion was calculated. This cycle (co-culture with fresh target cells) was repeated for up to three sequential stimulations to assess long-term proliferative capacity.

### Cytokine assay

To assess cytokine secretion by CAR-T cells, supernatants from cytotoxicity assays or mouse serum samples were collected. Concentrations of IL-2 (R&D Systems, DY202), IFN-γ (R&D Systems, DY285B), and IL-13 (R&D Systems, DY213) were determined by ELISA. To quantify IL-6 secretion *in vitro*, CAR-T cells (1×10^5^) were co-cultured with OVCAR3 (MSLN^+^) cells (1×10^5^) in 96-well plates. After 24 h of incubation at 37 °C in 5% CO_2_, culture supernatants were harvested, and IL-6 levels were measured by ELISA (R&D Systems, DY206).

### Membrane proteome array analysis

Membrane proteome array (MPA) analysis was conducted by Integral Molecular using a library of 6000 human membrane proteins individually overexpressed in live cells. Proteins were arrayed in duplicate in 384-well plates after 36 h of transfection. Prior to the analysis, the optimal concentration of the test antibody was determined on cells expressing a positive binding control (membrane-tethered Protein A) and a negative control (mock-transfected cells), followed by detection with a fluorescently labeled secondary antibody (Jackson ImmunoResearch, 109-606-008) via flow cytometry. The test antibody was then applied to the MPA at the predetermined concentration, and binding to the protein library was measured on an Intellicyt iQue flow cytometer by the same secondary antibody. Each array plate included positive (Fc-binding) and negative (empty vector) controls to ensure plate-to-plate reproducibility. Any off-target interactions identified by MPA were confirmed in a second flow cytometry experiment by serial dilutions of the test antibody, and the target identity was re-verified by sequencing. The complete MPA raw data, including screening thresholds, replicate concordance, and validation curves, are provided as **Supplementary Material**.

### SS1 and MOR-scFv affinity profiling by surface plasmon resonance

Recombinant human IgG1 Fc-tagged SS1-scFv and MOR-scFv were purchased from Biointron (Taizhou, China). The binding kinetics of each scFv against human and murine mesothelin (MSLN) were characterized by SPR using a Biacore 8K system (Cytiva). Each scFv-Fc fusion protein was captured on a Series Protein A chip (Cytiva) at densities of approximately 200 RU (SS1) or 200–275 RU (MOR), by injecting the respective scFv at 10 μL/min for 60 s. For binding studies, human and murine MSLN were injected at 30 μL/min over a range of concentrations: for SS1, 1.5625–50 nM (2-fold serial dilutions) with association and dissociation times of 120 s and 240 s, respectively; for MOR, 0.78125–50 nM (2-fold serial dilutions) with association and dissociation times of 120 s and 300 s, respectively. The chip surface was regenerated after each cycle using 10 mM Gly-HCl (pH 1.5) at 30 μL/min for 30 s. Kinetic parameters, including the equilibrium dissociation constant (KD), were derived from global fitting of both phases using a 1:1 binding model in Biacore Insight Evaluation software.

### Xenogeneic tumor model in NCG mice

All mouse experiments were performed in accordance with institutional guidelines (Youjiang Medical University for Nationalities, approval 2024111301 and 2025012301). For xenograft models, 5×10^6^ SKOV3 cells suspended in 200 μL of saline were subcutaneously injected into the right flank of 4 to 5-week-old female NCG mice (Jiangsu GemPharmatech). After implantation, mice were randomized into cohorts with equivalent tumor volumes (50–150 mm^3^). Tumor length and width were measured every 3–4 days with digital calipers, and tumor volume was calculated as V = ½ × length × width². Body weight was monitored every 3–4 days as an indicator of overall health. Daily clinical observations were performed, including assessment of activity, posture, and grooming. Mice were euthanized when the calculated tumor volume reached 2000 mm^3^, or when they met predefined humane endpoints (severe weight loss, moribund state, or inability to reach food/water). All cell doses were normalized to viable CAR-positive cells before injection. No anesthesia was used during tail-vein injections, as the procedure is rapid (completed within seconds) and anesthesia was not required by the institutional animal welfare guidelines; all injections were performed by trained personnel to minimize animal stress.

To evaluate the anti-tumor efficacy of the indicated CAR-T cells, on day 11 after tumor implantation, mice were injected via the tail vein with 1×10^6^ CAR-T cells per mouse (n = 7 per group): SS1-ϵζ-80BB, SS1-ϵζ-BB^ΔBRM^, SS1-TRuC, SS1-BBζ CAR-T and untransduced T cells.

To evaluate the dose-dependent toxicity of SS1-ϵζ-80BB CAR-T cells, a systematic dose-escalation study was conducted with 1×10^5^, 3×10^5^, 1×10^6^, and 5×10^6^ SS1-ϵζ-80BB CAR-T cells/mouse (n=8 per group).

To evaluate the toxicity of SS1-ϵζ-BB^ΔBRM^, SS1-ϵζ-80BB, and two 4-1BB-deleted variants (SS1-ϵζ-80^ECD^ and SS1-ϵζ-BB^ΔBRM^-80^ECD^), mice were injected via the tail vein on day 11 with 5×10^6^ CAR-T cells per mouse (n = 5–6 per group).

To assess the effect of scFv affinity on toxicity, mice were injected via the tail vein on day 11 with 5×10^6^ CAR-T cells per mouse using another donor. The experimental groups included the SS1-ϵζ-80BB, the MOR-ϵζ-80BB, the SS1-ϵζ-BB^ΔBRM^-80^ECD^ and the conventional CAR controls SS1-BBζ and SS1-TRuC CAR-T cells. For survival analysis, each group contained 5 mice (n = 5 per group). In addition, satellite groups of 3 mice each were included for the SS1-ϵζ-80BB and MOR-ϵζ-80BB cohorts to collect tissues at predefined time points.

### CAR DNA copies in mouse blood

To compare the expansion efficacy of different CAR constructs, mice were injected with 1×10^6^ CAR-T cells (SS1-ϵζ-80BB, SS1-ϵζ-BB^ΔBRM^, SS1-TRuC, SS1-BBζ, or untransduced T cells). Peripheral blood was collected from the tail vein of three mice per group. Erythrocytes were lysed using a commercial lysis buffer (Beyotime, C3702), and genomic DNA was extracted (Vazyme, 7E2403K4). Real-time PCR was performed on a 7500-Fast real-time PCR thermocycler (Applied Biosystems) by TaqMan probes targeting the WPRE sequence. Sample WPRE copy numbers per microgram of mouse blood DNA were calculated from the standard curve. The primer and probe sequences were as follows,

Forward primer: GCTATGTGGATACGCTGCTT;

Reverse primer: AGAGACAGCAACCAGGATTTA;

Probe: FAM-TCATGCTATTGCTTCCCGTATGGC-BHQ.

### Flow cytometric analysis of CAR-T cells *in vivo*

To compare the *in vivo* expansion of different CAR constructs, mice were injected with 1×10^6^ CAR-T cells (SS1-ϵζ-80BB, SS1-ϵζ-BB^ΔBRM^, SS1-TRuC, SS1-BBζ, or untransduced T cells). On days 30 and 35 post-infusion, 200 µL of whole blood was collected via retro-orbital bleeding and subjected to red blood cell lysis (Beyotime, C3702). Cells were stained with Brilliant Violet 785 anti-human CD45 (BioLegend, 304048) and PE/Cyanine7 anti-mouse CD45 (BioLegend, 103114). Stained samples were acquired on an ACEA Novocyte 3110 flow cytometer. Lymphocytes were gated by SSC-A versus FSC-A, and human T cells were defined as the human CD45^+^ and mouse CD45⁻ population.

On day 35, tumors were weighed. Tumors from two mice per group were pooled and enzymatically digested using the mouse tumor dissociation kit (Solarbio, P9000). The resulting cell suspensions were stained with the same antibodies, and Count Beads (BioLegend, 424902) were used to determine absolute human CD45^+^ T cell numbers by applying the identical gating strategy.

To compare the *in vivo* expansion of MOR-ϵζ-80BB and SS1-ϵζ-80BB, satellite mice were included in each group. Because the first deaths in the SS1-ϵζ-80BB cohort occurred on day 7 post-infusion, blood was collected on day 7 from three mice per group (satellite animals), and cells were stained with Brilliant Violet 785 anti-human CD45 and PE/Cyanine7 anti-mouse CD45. Human CD45^+^ T cells were analyzed using the same flow cytometric acquisition and gating procedures. Spleens were also collected from these mice on day 7, processed with the mouse tumor dissociation kit (Solarbio, P9000) to obtain cell suspensions, and stained with Brilliant Violet 785 anti-human CD45 and PE/Cyanine7 anti-mouse CD45. For subset analysis, CD4^+^ and CD8^+^ subsets were examined with FITC-anti-human CD4 (BioLegend, 344604) and BV510-anti-human CD8 (BioLegend, 344732), and memory/effector subsets (Tcm, Tem, Tscm, Tn, Teff) were examined with APC-anti-human CD45RO (BioLegend, 304210) using APC mouse IgG2a, κ isotype control (BioLegend, 400219), and AF488-anti-human CCR7 (BioLegend, 353206) using Alexa Fluor^®^ 488 mouse IgG2a, κ isotype control (BioLegend, 400233). All staining procedures were performed according to the manufacturers’ protocols. For markers with clearly separated positive and negative populations (CD4, CD8, and CAR detection), gating was based on natural population distribution. The gating strategy for splenic subset analysis is shown in **Supplementary Figure S7F**.

FSC-A vs SSC-A → Lymphocytes → Mouse CD45-and Human CD45^+^ → CD4/CD8.

FSC-A vs SSC-A → Lymphocytes → Mouse CD45-and Human CD45^+^ → CD45RO/CCR7.

### Histological staining

Immunohistochemistry was performed by Nanjing Youmeng Biotechnology Co., Ltd. Formalin-fixed, paraffin-embedded tissue sections were deparaffinized in xylene and rehydrated through a graded ethanol series. Antigen retrieval was performed by microwaving sections in EDTA buffer (pH 8.0). Endogenous peroxidase activity was blocked with 3 % H_2_O_2_ for 25 min at room temperature. Sections were then blocked with 3 % BSA for 30 min and incubated with anti-CD3 antibody (YM-E00005, diluted in PBS) overnight at 4 °C. An HRP-conjugated secondary antibody was applied to the sections for 50 min at room temperature. DAB (3,3′-diaminobenzidine) substrate was then used to develop the staining, followed by hematoxylin counterstaining, dehydration, and mounting. Images were captured with a light microscope. Whole-slide IHC quantification was performed using HALO software (Indica Labs, USA) to quantify positive cell number, positive area, average optical density, and total tissue area, allowing calculation of positive cell percentage, positive cell density, and positive area ratio.

H&E staining was conducted by Nanjing Youmeng Biotechnology Co., Ltd. Formalin-fixed, paraffin-embedded tissue sections were deparaffinized in xylene and rehydrated through a graded ethanol series to distilled water. Specimens were stained with hematoxylin for 3–5 min, rinsed in tap water, and treated with bluing reagent. Dehydration was carried out in 85 % and 95 % ethanol (4 min each), then stained with eosin for 10 s. Complete dehydration was achieved in pure ethanol, followed by xylene clearing and mounting with neutral resin. All images were acquired on a light microscope. H&E sections were evaluated by blinded histopathological scoring performed by pathologists.

### Statistical analysis

For donor-matched two-group comparisons, a two-tailed paired t-test was used. For two-group comparisons of independent samples, a two-tailed unpaired t-test was used. For comparisons involving more than two groups with repeated measures from the same donor, repeated-measures one-way ANOVA with appropriate post-hoc corrections (Tukey’s or Dunnett’s) was applied. For longitudinal data (tumor growth curves, serial killing assays, cumulative expansion curves), two-way repeated-measures ANOVA or mixed-effects models with group-by-time interaction and multiplicity correction were used. Mouse overall survival was estimated with the log-rank (Mantel-Cox) test. Technical replicates were averaged within each independent donor or experiment before analysis. For experiments with fewer than three independent donors, data are presented as descriptive trends without statistical inference. For single-donor experiments with repeated measurements over time (e.g., proliferation assays), two-way repeated-measures ANOVA was used descriptively; no formal hypothesis testing was applied. All other single-donor experiments are presented as exploratory observations. GraphPad Prism 8.3.0 was used for statistical analysis and graphing, and SnapGene 6.0.2 was used for DNA sequence analysis. Data are presented as mean ± SD unless otherwise indicated. *P* < 0.05 was considered statistically significant.

## Data Availability

The datasets presented in this study can be found in online repositories. The names of the repository/repositories and accession number(s) can be found in the article/**Supplementary Material**.

## References

[1] De Marco RC Monzo HJ Ojala PM . CAR T cell therapy: a versatile living drug. Int J Mol Sci. (2023) 24(7):6300. doi: 10.3390/ijms24076300 37047272 PMC10094630

[2] Cappell KM Kochenderfer JN . Long-term outcomes following CAR T cell therapy: what we know so far. Nat Rev Clin Oncol. (2023) 20:359–71. doi: 10.1038/s41571-023-00754-1 37055515 PMC10100620

[3] Helsen CW Hammill JA Lau VWC Mwawasi KA Afsahi A Bezverbnaya K . The chimeric TAC receptor co-opts the T cell receptor yielding robust anti-tumor activity without toxicity. Nat Commun. (2018) 9:3049. doi: 10.1038/s41467-018-05395-y 30076299 PMC6076291

[4] Xu Y Yang Z Horan LH Zhang P Liu L Zimdahl B . A novel antibody-TCR (AbTCR) platform combines Fab-based antigen recognition with gamma/delta-TCR signaling to facilitate T-cell cytotoxicity with low cytokine release. Cell Discov. (2018) 4:62. doi: 10.1038/s41421-018-0066-6 30479831 PMC6242878

[5] Baeuerle PA Ding J Patel E Thorausch N Horton H Gierut J . Synthetic TRuC receptors engaging the complete T cell receptor for potent anti-tumor response. Nat Commun. (2019) 10:2087. doi: 10.1038/s41467-019-10097-0 31064990 PMC6504948

[6] Lesch S Nottebrock A Rataj F Heise C Endres S Kobold S . PD-1-CD28 fusion protein strengthens mesothelin-specific TRuC T cells in preclinical solid tumor models. Cell Oncol. (2022) 46:227–35. doi: 10.1007/s13402-022-00747-9 36409438 PMC9947055

[7] Liu Y Liu G Wang J Zheng Z-Y Jia L Rui W . Chimeric STAR receptors using TCR machinery mediate robust responses against solid tumors. Sci Transl Med. (2021) 13(586):eabb5191. doi: 10.1126/scitranslmed.abb5191 33762437

[8] Dobrin A Lindenbergh PL Shi Y Perica K Xie H Jain N . Synthetic dual co-stimulation increases the potency of HIT and TCR-targeted cell therapies. Nat Cancer. (2024) 5:760–73. doi: 10.1038/s43018-024-00744-x 38503896 PMC11921049

[9] Harding FA McArthur JG Gross JA Raulet DH Allison JP . CD28-mediated signalling co-stimulates murine T cells and prevents induction of anergy in T-cell clones. Nature. (1992) 356:607–9. doi: 10.1038/356607a0 1313950

[10] Burton J Siller-Farfan JA Pettmann J Salzer B Kutuzov M van der Merwe PA . Inefficient exploitation of accessory receptors reduces the sensitivity of chimeric antigen receptors. Proc Natl Acad Sci USA. (2023) 120:e2216352120. doi: 10.1073/pnas.2216352120 36598945 PMC9926289

[11] Malissen B Bongrand P . Early T cell activation: integrating biochemical, structural, and biophysical cues. Annu Rev Immunol. (2015) 33:539–61. doi: 10.1146/annurev-immunol-032414-112158 25861978

[12] Guedan S Calderon H Posey ADJ Jr. Maus MV . Engineering and design of chimeric antigen receptors. Mol Ther Methods Clin Dev. (2019) 12:145–56. doi: 10.1016/j.omtm.2018.12.009 30666307 PMC6330382

[13] Feucht J Sun J Eyquem J Ho YJ Zhao Z Leibold J . Calibration of CAR activation potential directs alternative T cell fates and therapeutic potency. Nat Med. (2019) 25:82–8. doi: 10.1038/s41591-018-0290-5 30559421 PMC6532069

[14] Mog BJ Marcou N DiNapoli SR Pearlman AH Nichakawade TD Hwang MS . Preclinical studies show that Co-STARs combine the advantages of chimeric antigen and T cell receptors for the treatment of tumors with low antigen densities. Sci Transl Med. (2024) 16:eadg7123. doi: 10.1126/scitranslmed.adg7123 38985855 PMC12226805

[15] Wang J Zhang X Zhou Z Liu Y Yu L Jia L . A novel adoptive synthetic TCR and antigen receptor (STAR) T-cell therapy for B-cell acute lymphoblastic leukemia. Am J Hematol. (2022) 97(8):992–1004. doi: 10.1182/blood-2020-136833 35491511

[16] Zhang Z Liu C Wang M Sun R Yang Z Hua Z . Treating solid tumors with TCR-based chimeric antigen receptor targeting extra domain B-containing fibronectin. J Immunother Cancer. (2023) 11(8):e007199. doi: 10.1136/jitc-2023-007199 37586774 PMC10432677

[17] Burgess SJ Stauss HJ Morris EC . Chimeric CD3ζ chains containing CD28 signalling motifs enhance antigen-specific IL-2 production and expansion of human TCR-engineered T cells *in vitro*. Immunother Adv. (2026) 6:ltaf038. doi: 10.1093/immadv/ltaf038 41930278 PMC13042253

[18] Webb L Lofgren M Patterson T Watt A Lajoie J Zieba A . Membrane-bound IL-15 co-expression powers a potent and persistent CD70-targeted TRuC T-cell therapy. Front Immunol. (2025) 16. doi: 10.3389/fimmu.2025.1609658 40519930 PMC12162932

[19] Li M Zheng R Liu Z Zhang P Zhu T Xin X . Optimized BCMA/CS1 bispecific TRuC-T cells secreting IL-7 and CCL21 robustly control multiple myeloma. Front Immunol. (2024) 15. doi: 10.3389/fimmu.2024.1502936 39776916 PMC11703830

[20] McCarthy D Lofgren M Watt A Horton H Kieffer-Kwon P Ding J . Functional enhancement of mesothelin-targeted TRuC-T cells by a PD1-CD28 chimeric switch receptor. Cancer Immunol Immunother. (2023) 72:4195–207. doi: 10.1007/s00262-023-03556-7 37848682 PMC10700406

[21] Lah S Kim S Kang I Kim H Hupperetz C Jung H . Engineering second-generation TCR-T cells by site-specific integration of TRAF-binding motifs into the CD247 locus. J Immunother Cancer. (2023) 11(4):e005519. doi: 10.1136/jitc-2022-005519 37019470 PMC10083865

[22] Call ME Wucherpfennig KW . The T cell receptor: critical role of the membrane environment in receptor assembly and function. Annu Rev Immunol. (2005) 23:101–25. doi: 10.1146/annurev.immunol.23.021704.115625 15771567

[23] Xin W Huang B Chi X Liu Y Xu M Zhang Y . Structures of human gammadelta T cell receptor-CD3 complex. Nature. (2024) 630:222–9. doi: 10.1038/s41586-024-07439-4 PMC11153141 38657677

[24] Blumberg RS Ley S Sancho J Lonberg N Lacy E Mcdermott F . Structure of the T-cell antigen receptor: evidence for two CD3 ϵ subunits in the T-cell receptor-CD3 complex. PNAS. (1990) 87:7220–4. doi: 10.1073/pnas.87.18.7220 2144901 PMC54715

[25] Goff SL Johnson LA Black MA Xu H Zheng Z Cohen CJ . Enhanced receptor expression and *in vitro* effector function of a murine-human hybrid MART-1-reactive T cell receptor following a rapid expansion. Cancer Immunol Immunother. (2010) 59:1551–60. doi: 10.1007/s00262-010-0882-5 20628878 PMC3436071

[26] Viola A Lanzavecchia A . T cell activation determined by T cell receptor number and tunable thresholds. Sci (New York NY). (1996) 273:104–6. doi: 10.1126/science.273.5271.104 8658175

[27] Ahmadi M King JW Xue SA Voisine C Holler A Wright GP . CD3 limits the efficacy of TCR gene therapy *in vivo*. Blood. (2011) 118:3528–37. doi: 10.1182/blood-2011-04-346338 21750319

[28] Baniyash M . TCR zeta-chain downregulation: curtailing an excessive inflammatory immune response. Nat Rev Immunol. (2004) 4:675–87. doi: 10.1038/nri1434 15343367

[29] Degirmencay A Thomas S Holler A Burgess S Morris EC Stauss HJ . Exploitation of CD3zeta to enhance TCR expression levels and antigen-specific T cell function. Front Immunol. (2024) 15:1386132. doi: 10.3389/fimmu.2024.1386132 38873603 PMC11169823

[30] Nam KO Shin SM Lee HW . Cross-linking of 4-1BB up-regulates IL-13 expression in CD8(+) T lymphocytes. Cytokine. (2006) 33:87–94. doi: 10.1016/j.cyto.2005.12.003 16488622

[31] Call ME Pyrdol J Wiedmann M Wucherpfennig KW . The organizing principle in the formation of the T cell receptor-CD3 complex. Cell. (2002) 111:967–79. doi: 10.1016/s0092-8674(02)01194-7 12507424 PMC3420808

[32] Guo H Qian L Cui J . Focused evaluation of the roles of macrophages in chimeric antigen receptor (CAR) T cell therapy associated cytokine release syndrome. Cancer Biol Med. (2021) 19:333–42. doi: 10.20892/j.issn.2095-3941.2021.0087 34570442 PMC8958886

[33] Hao Z Li R Meng L Han Z Hong Z . Macrophage, the potential key mediator in CAR-T related CRS. Exp Hematol Oncol. (2020) 9:15. doi: 10.1186/s40164-020-00171-5 32665874 PMC7349474

[34] Sachdeva M Duchateau P Depil S Poirot L Valton J . Granulocyte-macrophage colony-stimulating factor inactivation in CAR T-cells prevents monocyte-dependent release of key cytokine release syndrome mediators. J Biol Chem. (2019) 294:5430–7. doi: 10.1074/jbc.ac119.007558 30804212 PMC6462525

[35] Sterner RC Sterner RM . CAR-T cell therapy: current limitations and potential strategies. Blood Cancer J. (2021) 11:69. doi: 10.1038/s41408-021-00459-7 33824268 PMC8024391

[36] Sterner RM Sakemura R Cox MJ Yang N Khadka RH Forsman CL . GM-CSF inhibition reduces cytokine release syndrome and neuroinflammation but enhances CAR-T cell function in xenografts. Blood. (2019) 133:697–709. doi: 10.1182/blood-2018-10-881722 30463995 PMC6376281

[37] Yang S Xu J Dai Y Jin S Sun Y Li J . Neutrophil activation and clonal CAR-T re-expansion underpinning cytokine release syndrome during ciltacabtagene autoleucel therapy in multiple myeloma. Nat Commun. (2024) 15:360. doi: 10.1038/s41467-023-44648-3 38191582 PMC10774397

[38] Haas AR Tanyi JL O'Hara MH Gladney WL Lacey SF Torigian DA . Phase I study of lentiviral-transduced chimeric antigen receptor-modified T cells recognizing mesothelin in advanced solid cancers. Mol Ther. (2019) 27:1919–29. doi: 10.1016/j.ymthe.2019.07.015 31420241 PMC6838875

[39] Ko AH Jordan AC Tooker E Lacey SF Chang RB Li Y . Dual targeting of mesothelin and CD19 with chimeric antigen receptor-modified T cells in patients with metastatic pancreatic cancer. Mol Ther. (2020) 28:2367–78. doi: 10.1016/j.ymthe.2020.07.017 32730744 PMC7647666

[40] Yue YL Liu JJ Ma H Pan ZD Wang L Zhang JW . Generating potent and persistent antitumor immunity via affinity-tuned CAR-T cells targeting mesothelin. Acta Pharmacol Sin. (2025) 46(10):2849–62. doi: 10.1038/s41401-025-01572-0 40341217 PMC12460624

[41] Yang Y Vedvyas Y Alcaina Y Trumper SJ Babu DS Min IM . Affinity-tuned mesothelin CAR T cells demonstrate enhanced targeting specificity and reduced off-tumor toxicity. JCI Insight. (2024) 9(22):e186268. doi: 10.1172/jci.insight.186268 39576012 PMC11601908

