# Supplementary material for: Affinity and costimulatory architecture-associated toxicity in mesothelin-targeted TCR-mimetic CAR-T cells
Source: Front Immunol. 2026 Sep 24;17:1947351. doi: 10.3389/fimmu.2026.1947351 (PMC13646116; doi:10.3389/fimmu.2026.1947351)
Supplement: Supplementary file 1 [file SupplementaryFile1.docx]

Supplementary material

**This PDF file includes：**

Supplemental Figure.S1 to Figure.S13

**Contents**

**Supplemental FIGURE 1** Cytotoxicity of SS1-εζ-BB^ΔBRM^ TCR-mimetic CAR-T.

**Supplemental FIGURE 2** In vitro immuno-function with CD3ζ overexpression.

**Supplemental FIGURE 3** SS1-εζ-80BB TCR-mimetic CAR-T cells show robust expansion and elevated IL-6 secretion.

**Supplemental FIGURE 4** In vivo TCR-mimetic CAR-T cell expansion and tumor infiltration.

**Supplemental FIGURE 5** SS1-εζ-80BB TCR-mimetic CAR-T cells displayed dose-dependent toxicity in NCG mice bearing SKOV3 tumors.

**Supplemental FIGURE 6**  Membrane protein array of SS1-scFv and SPR affinity for MSLN.

**Supplemental FIGURE 7** Deletion of the 4-1BB intracellular domain from 80BB abolishes in vivo toxicity.

**Supplemental FIGURE 8** SPR affinity assessment of MOR scFv for human and murine MSLN.

**Supplemental FIGURE 9** Comparison of SS1- and MOR‑based 80BB TCR‑mimetic CAR-T cells in vitro and in vivo.

**Supplemental FIGURE 10** Characteristics of MOR-εζ-80BB and SS1-εζ-80BB TCR-mimetic CAR-T cells in the blood and spleen.

**Supplemental FIGURE 11** Human T cell infiltration and histopathology in the lung and liver.

**Supplemental FIGURE 12**  Histopathological analysis of liver, spleen, and lung by H&E staining.

**Supplemental FIGURE 13**  Semi-quantitative histopathological scoring of liver, spleen, and lung.

**FIGURE S1** Cytotoxicity of SS1-εζ-BB^ΔBRM^-TCR-mimetic CAR-T. **(A-C)** Representative flow cytometry plots showing CAR expression on CD3⁺ T cells transduced with SS1‑εζ, SS1‑εζ‑BB, or SS1‑εζ‑BB^ΔBRM^ in three different healthy donors. **(D)** Validation of MSLN surface overexpression in SKOV3 and OVCAR3 cells by flow cytometry. **(E)** Cytotoxicity of Mock T, SS1‑εζ, SS1‑εζ‑BB, and SS1‑εζ‑BB^ΔBRM^ T‑CAR‑T cells against MSLN⁺ SKOV3-luc cells at an E:T ratio of 2:1. Data are from three independent donors, each shown as a single data point after averaging technical replicates (n = 3). *p* values were calculated by one‑way ANOVA. All error bars denote SD.

**FIGURE S2** In vitro immuno-function with CD3ζ overexpression. **(A-B)** Flow cytometry plots showing CAR and CD80 surface co‑expression on CD3⁺ T cells transduced with SS1‑ε(TRuC), SS1-ε-80BB, SS1-εζ-80BB, SS1-εδ-80BB, and SS1-εγ-80BB from two independent donors. **(C)** Cytotoxicity of the CAR-T cells against MSLN⁺ SKOV3-luc cells at an E:T ratio of 1:1. Data are from two independent donors, each shown as a single data point after averaging technical replicates (n = 2). Data are shown as descriptive trends; no statistical inference was performed. All error bars denote SD. **(D)** IL-2 concentrations in the supernatant 24 h after co‑culture with SKOV3-luc cells at an E:T ratio of 1:1.Data are from two independent donors, each shown as a single data point after averaging technical replicates (n = 2). Data are shown as descriptive trends; no statistical inference was performed. All error bars denote SD. **(E, F)** IFN-γ (E) and IL-2 (F) secretion from SS1‑ε and SS1‑εζ CAR‑T cells after 24 h of co‑culture with SKOV3-luc cells (E:T = 1:1). Data are from three independent donors, each shown as a single data point after averaging technical replicates (n = 3). *p* values were calculated by paired t‑test. All error bars denote SD.

**FIGURE S3** SS1-εζ-80BB TCR-mimetic CAR-T cells show robust expansion and elevated IL-6 secretion. **(A)** CAR‑positive cells were prepared and verified by flow cytometry for animal experiments. The constructs tested included SS1‑εζ‑80BB, SS1‑εζ‑BB^ΔBRM^, SS1‑TRuC, SS1‑BBζ, or untransduced T cells (mock). (**B)** CAR DNA copies in peripheral blood measured by qPCR at the indicated time points. Data are from three biologically independent mice (n = 3). **(C)** IL‑6 concentrations in the supernatant 24 h after co‑culture with MSLN⁺ OVCAR3 cells at an E:T ratio of 1:1. Data are from two independent donors, each shown as a single data point after averaging technical replicates (n = 2). Data are shown as descriptive trends; no statistical inference was performed. Data are presented as mean ± SD. **(D)** Body weight changes of mice in each group over time.

**FIGURE S4** In vivo TCR-mimetic CAR-T cell expansion and tumor infiltration.

**(A)** Representative flow cytometry plots of peripheral blood CD45⁺ cells on days 30 and 35, this is an exploratory analysis and no statistical inference was performed. **(B)** Quantification of tumor-infiltrating human CD45⁺ cells by counting beads assay on day 35. CAR-T cells were pooled from tumors of two mice per experimental group for analysis; this is an exploratory analysis and no statistical inference was performed.

**FIGURE S5**  SS1-εζ-80BB TCR-mimetic CAR-T cells displayed dose-dependent toxicity in NCG mice bearing SKOV3 tumors. **(A)** CAR‑positive cells were prepared and verified by flow cytometry for animal experiments. **(B)** Body weight changes of mice in the 1×10⁵, 3×10⁵, and 1×10⁶ dose groups over time. Bold black lines indicate mice that succumbed.

**FIGURE S6** Membrane protein array of SS1-scFv and SPR affinity for MSLN. **(A–C)** Membrane protein array. **(A)** Representative membrane protein array image; arrows indicate FCGR1A (Fc gamma receptor Ia) and TMEM43 (transmembrane protein 43). **(B)** Fluorescence intensity of SS1-scFv binding to membrane proteins at concentrations ranging from 0.3 to 20 ug/mL. **(C)** Fluorescence intensity of SS1-scFv binding to intracellular proteins at the same concentration range. **(D, E)** Surface plasmon resonance analysis of SS1 binding affinity to human MSLN (D) and murine MSLN (E).

**FIGURE S7** Deletion of the 4-1BB intracellular domain from 80BB abolishes in vivo toxicity. **(A)** CAR‑positive cells were prepared and verified by flow cytometry for animal experiments. The constructs tested included: SS1‑εζ‑BB^ΔBRM^, SS1‑εζ‑80BB, SS1‑εζ‑80^ECD^, and SS1‑εζ‑BB^ΔBRM^‑80^ECD^. **(B)** Body weight changes of mice in the 5×10⁶ dose group over time. Bold black lines indicate mice that succumbed.

**FIGURE S8** SPR affinity assessment of MOR scFv for human and murine MSLN. **(A, B)** Surface plasmon resonance assessment of MOR binding affinity to human MSLN **(A)** and murine MSLN **(B)**.

**FIGURE S9** Comparison of SS1- and MOR‑based 80BB TCR‑mimetic CAR-T cells in vitro and in vivo. **(A)** CAR‑positive cells were prepared and verified by flow cytometry for animal experiments. The constructs tested included: MOR-εζ-80BB, SS1-εζ-80BB, SS1‑εζ‑BB^ΔBRM^-80^ECD^, SS1-TRuC, SS1-BBζ (conventional 4-1BBζ CAR) and Mock T cells. **(B, C)** IFN‑γ (B) and IL‑2 (C) concentrations in the supernatant 24 h after co‑culture with MSLN⁺ SKOV3 cells at an E:T ratio of 1:1. Data are from three technical replicates from one representative donor (mean ± SD) and are shown for illustrative purposes; no statistical inference was performed. These in vitro data were generated using the same CAR‑T cell preparation subsequently used for the in vivo experiments, allowing direct comparison of in vitro functionality with in vivo outcomes. **(D)** IL‑6 concentrations in the supernatant 24 h after co‑culture with MSLN⁺ OVCAR3 cells at an E:T ratio of 1:1. Data are from three technical replicates from one representative donor and are shown for illustrative purposes; no statistical inference was performed. **(E)** Body weight changes of mice in the 5×10⁶ dose group over time.

**FIGURE S10** Characteristics of MOR-εζ-80BB and SS1-εζ-80BB TCR-mimetic CAR-T cells in the blood and spleen. **(A)** Flow cytometry plots of human CD45⁺ lymphocytes in peripheral blood of MOR‑εζ‑80BB and SS1‑εζ‑80BB satellite mice on day 7, when SS1‑εζ‑80BB showed lethal toxicity. Plots from all three individual mice per group are shown. **(B)** Flow cytometry plots of human CD4⁺ and CD8⁺ T cells in the spleen on day 7. Plots from all three individual mice per group are shown. **(C)** Flow cytometry plots of splenic T cell differentiation phenotypes based on CD45RO and CCR7 expression on day 7. Plots from all three individual mice per group are shown. **(D)** Quantification of CD4⁺ and CD8⁺ T cell percentages among splenic human CD45⁺ T cells from the flow cytometry data shown in (B). Data are from three biologically independent mice (n = 3). **(E)** Quantification of T cell subset percentages among splenic human CD45⁺ T cells from the flow cytometry data shown in (C). Data are from three biologically independent mice (n = 3). Defined as: Tcm (CD45RO⁺CCR7⁺), Tem (CD45RO⁺CCR7⁻), Tn+Tscm (CD45RO⁻CCR7⁺), and Teff (CD45RO⁻CCR7⁻). Data are from three biologically independent mice (n = 3). *p* values were calculated by paired t‑test. All error bars denote SD.

**FIGURE S11** Human T cell infiltration and histopathology in the lung and liver. **(A)** IHC analysis of human CD3⁺ T cell infiltration in the lung and liver. Images are from two individual mice per group; brown staining denotes human CD3⁺ cells. **(B)** Whole‑slide quantification of human CD3⁺ T cell infiltration in the lung and liver based on IHC analysis of entire tissue sections. Positive cell density (cells/mm²) are shown for each individual mouse. **(C)** Summary of positive cell count, tissue area, and positive cell density in the lung and liver for human CD3⁺ cells.

**FIGURE S12** Histopathological analysis of liver, spleen, and lung by H&E staining. **(A)** H&E-stained sections of liver, spleen, and lung from satellite mice euthanized on day 7 (two mice per group: SS1-εζ-80BB and MOR-εζ-80BB) and from one mouse that died early with minimal tumor burden (SS1-εζ-80BB found dead). Arrow annotations: liver (black, hepatocellular edema; red, inflammatory infiltration; yellow, fibrous hyperplasia; green, focal necrosis); spleen (black, capsular hyperplasia; red, white pulp atrophy; yellow, granulocyte infiltration; green, red pulp congestion); lung (black, alveolar wall thickening; red, atypical cell clusters; yellow, inflammatory infiltration; green, bronchial luminal eosinophilic material).

**FIGURE S13** Semi‑quantitative histopathological scoring of liver, spleen, and lung. **(A)** Four‑grade histopathological scoring system (0–4) used for evaluating tissue sections. **(B)** Histopathological scores for liver: cellular edema, inflammatory cell infiltration, fibrous hyperplasia, fatty degeneration, congestion, and necrosis. **(C)** Histopathological scores for spleen: capsular hyperplasia, white pulp atrophy, inflammatory cell infiltration, and red pulp congestion. **(D)** Histopathological scores for lung: alveolar wall thickening, atypical cell clusters, inflammatory cell infiltration, and congestion. Scores were assigned by blinded histopathological evaluation performed by qualified pathologists. SS1‑εζ‑80BB‑found dead within 24 h represents mice that died early with minimal tumor burden; histopathological data for this group reflect terminal organ damage. Data are shown as individual scores for each mouse; SS1‑εζ‑80BB (n = 2), MOR‑εζ‑80BB (n = 2), and SS1‑εζ‑80BB found dead within 24 h (n = 1).
